# Heavy atom oriented orbital angular momentum manipulation in metal-free organic phosphors†

**DOI:** 10.1039/d1sc05689a

**Published:** 2021-12-24

**Authors:** Wenhao Shao, Hanjie Jiang, Ramin Ansari, Paul M. Zimmerman, Jinsang Kim

**Affiliations:** Department of Chemistry, Department of Chemical Engineering, Department of Materials Science and Engineering, Macromolecular Science and Engineering, University of Michigan Ann Arbor Michigan 48109 USA paulzim@umich.edu jinsang@umich.edu

## Abstract

Metal-free purely organic phosphors (POPs) are emerging materials for display technologies, solid-state lighting, and chemical sensors. However, due to limitations in contemporary design strategies, the intrinsic spin–orbit coupling (SOC) efficiency of POPs remains low and their emission lifetime is pinned in the millisecond regime. Here, we present a design concept for POPs where the two main factors that control SOC—the heavy atom effect and orbital angular momentum—are tightly coupled to maximize SOC. This strategy is bolstered by novel natural-transition-orbital-based computational methods to visualize and quantify angular momentum descriptors for molecular design. To demonstrate the effectiveness of this strategy, prototype POPs were created having efficient room-temperature phosphorescence with lifetimes pushed below the millisecond regime, which were enabled by boosted SOC efficiencies beyond 10^2^ cm^−1^ and achieved record-high efficiencies in POPs. Electronic structure analysis shows how discrete tuning of heavy atom effects and orbital angular momentum is possible within the proposed design strategy, leading to a strong degree of control over the resulting POP properties.

## Abstract 2

A new design concept for metal-free organic phosphors is proposed to tightly couple the heavy atom effect with changes in orbital angular momentum. Bright phosphors were created with record-fast lifetimes below the millisecond regime.

## Graphical abstract

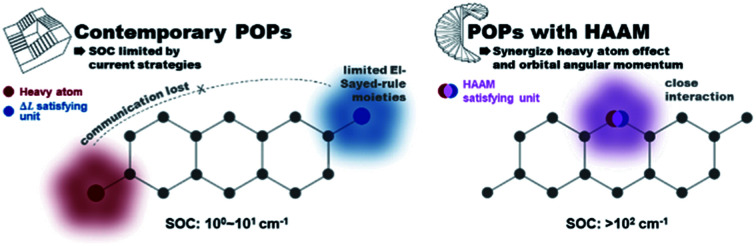

## 1. Introduction

**Fig. 1:**
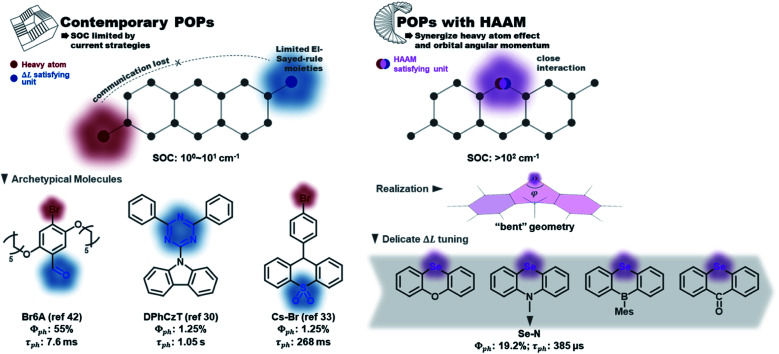
Contemporary POP design *vs.* POPs designed with the heavy atom oriented angular momentum manipulation (HAAM) concept implemented.

Organic phosphors are the functional components in modern technologies such as displays, solid-state lighting, and chemical sensors. While conventional organometallic phosphors suffer from metal ion dislocation,^1,2^ device longevity problems, and toxicity issues, metal-free purely organic phosphors (POPs) have many advantageous properties such as large design windows, easy processability, economic material cost, and lower toxicity.^3–5^ However, POPs typically exhibit long emission lifetimes due to the involvement of spin-forbidden transitions, which should be facilitated by spin–orbit coupling (SOC).^6^ In the design of contemporary POPs, SOC is promoted mostly by heavy atom effects and the El-Sayed rule. Fig. 1 listed a few representative POPs with halogen or chalcogen heavy atoms: Br,^7–19^ I,^20–22^ or Se.^23–28^ The El-Sayed rule explains the necessity of orbital angular momentum change in promoting SOC,^29^ and the utilization of carbonyl,^7,8,10–12,14,30,31^ heterocyclic rings^32–34^ (*e.g.* triazine in DPhCzT^32^), and other moieties having rich non-bonding electrons (*e.g.* sulfoxide in Cs–Br^35^) to stimulate low energy (n,π*) states and thus create (π,π*)–(n,π*) transition channels. Due to the forbidden nature of singlet–triplet transitions and consequently slow triplet emission, matrix engineering to effectively suppress collisional quenching (the major non-radiative decay route) is essential to achieve POPs with decent quantum efficiency.^36^ For that purpose, crystal design and strong intermolecular bonding between POPs and rigid matrix have been implemented.^10,30,35,37–44^ The slow decay nature of POPs, on the other hand, has been explored to create persistent emitters with long lifetimes in the 10^−1^ to 10^0^ second regime.^3^

However, current design strategies have reached their limit. The intrinsic SOC efficiencies of POPs remain low (*e.g.* T_1_–S_0_ SOC is usually 10^0^ to 10^1^ cm^−1^) with their emission lifetime stuck in the millisecond to second regime. The lack of synergistic interactions between the SOC-promoting components is the major deficiency of contemporary design strategies. On one hand, the El-Sayed rule hasn't been fully exploited to capture the detailed picture of molecular features leading to angular momentum changes between spin states. On the other hand, the interplay between the heavy atom effect and orbital angular momentum is missing, *i.e.*, heavy atoms are not rationally positioned in such a way that their orbitals strongly interact with those undergoing angular momentum change. Such delicate manipulations are not required in organometallic complexes, where the angular momentum changes are centered on the metal atom. In POPs, however, this connection is not trivial to achieve but could have a great impact on increasing SOCs. An additional factor that has hindered creation of better design strategies is that contemporary computational tools—while able to quantify SOCs—have provided little insight into the electronic origins of the SOC within chromophores.

Here, we report a novel molecular design concept to manipulate SOC in POPs that synergistically uses the heavy atom effect in close connection with orbital angular momentum to overcome SOC limits of organic phosphors. To emphasize the core of our design concept that uses heavy atoms to directly stimulate Δ*L*, we named it “heavy atom oriented orbital angular momentum manipulation” (HAAM) (see Fig. 1). This rational design strategy enhances SOCs to over 10^2^ cm^−1^ and pushes the lifetime limit of organic phosphors to below ms regime, as demonstrated in a series of prototype POPs using chalcogen heavy atoms. HAAM is confirmed to be operational through a novel natural transition orbital (NTO)-based computational method to visualize the molecular orbital origins of SOC. In total, this work introduces the HAAM design concept and tests its relevance using theory, computation, and experiment.

## 2. Results

### 2.1. Theory behind SOC

Before moving to the molecular design, it's worth revisiting the theory behind SOC that motivates the HAAM concept. Under Fermi's golden rule, the transition rate (state 1 to state 2) for intersystem crossing is given as *k*_12_ ∝ |*ψ*_2_|*H*_SO_|*ψ*_1_|^2^*ρ*(*E*_12_).^45^ Here, *ρ*(*E*_12_) denotes the joint density of states of the initial and final wavefunctions, *ψ*_1_ and *ψ*_2_; *H*_SO_ is the transition Hamiltonian, and the term *ψ*_2_|*H*_SO_|*ψ*_1_ is the SOC transition matrix element (SOCME). For one-electron systems under relativistic conditions, the main-part of the spin–orbit Hamiltonian is 
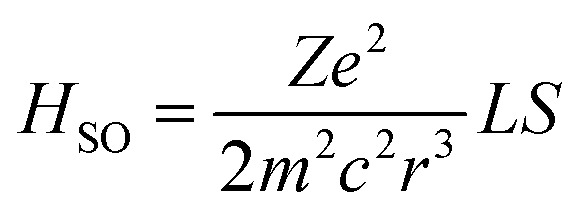
,^46^ where *r* is the orbital radius, *L* and *S* are the orbital and spin angular momentum, respectively. For many-electron systems, the Hamiltonian is expressed in terms of Breit-Pauli (BP) Hamiltonian^47^ as1
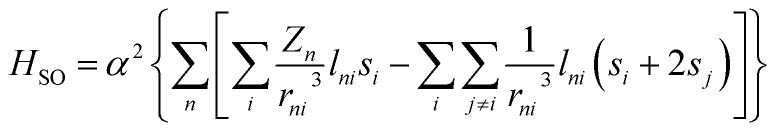
where the first term represents one-electron contributions involving electrons (*i*) and atoms (*n*), and the second represents two-electron (*i* and *j*) interactions. Here *α* is the fine structure constant; *Z*_*n*_ the atomic number of atom *n*; *r*_*ni*_ is the electron–nuclear distance, *l*_*ni*_ the orbital angular momentum; and *s*_*i*_ is the spin angular momentum. In one electron situation, since the expectation value of *r*^−3^ is proportional to *Z*^3^, SOCME scales with *Z*^4^. The scaling factor is more complicated in multi-electron systems due to the screening effects of core electrons, but overall, SOCME dramatically increases with heavy elements.

Intuitively, the coupling of electrons' spin and angular momentums in eqn (1) follows the conservation of total angular momentum rule—that since *S* changes with a spin change, the orbital angular momentum, *L*, has to change as well. The El-Sayed rule^29^ qualitatively elucidated the angular momentum conservation law, for example in a typical case the (π,π*)–(n,π*) transition carries a change in orbital angular momentum (Δ*L*). On the other hand, large Δ*L* has been observed in other types of transitions,^24,48^ even for (π,π*)–(π,π*) transitions.

More importantly, it is clear from eqn (1) that orbital and spin angular momenta are coupled through the interaction with atomic number. Importantly, due to the form of eqn (1), the heavy atom must be in close proximity with the spin orbital transition, otherwise the effective *Z* from that atom is suppressed by the *r*^−3^ dependence of *H*_SO_. While our discussion here makes these principles transparent, in practice the combination of heavy atom effects with orbital angular momentum change has rarely been addressed in the design of POPs. For instance, Sarkar and Hendrickson *et al.*^49^ examined benzaldehydes with Br substitution at various positions and revealed extraordinarily boosted SOC when Br is at the *para* position relative to aldehyde. However, the orbital angular momentum influence was not discussed although it was clear from their results that Br's contribution to Δ*L* was drastically enhanced at the *para* position. In our recent reports,^24,48^ we elucidated the origin of efficient SOC from the perspective of Δ*L* and expanded the Δ*L*-promoting mechanism beyond traditional (π,π*)–(n,π*) transitions, but the interaction of heavy atom with Δ*L* was not reviewed. In short, HAAM strategy hasn't been well established in the systematic molecular design of POPs, which gives an open design space to explore the HAAM method for creating novel, tunable organic triplet emitters.

To help instantiate the HAAM strategy, *ab initio* simulations can provide insight into proposed chromophore designs by revealing their specific electronic structures and SOC elements. In particular, the restricted active space-spin flip (RAS-SF) method^50–54^ is a wave function theory that is well-suited for treating electronically excited states^55,56^ of photoactive molecular systems. RAS-SF has been shown to accurately treat a variety of challenging electronic structure problems, including polyradicals, singlet fission mechanisms, and charge transfer processes.^57–61^ Recent work has enabled RAS-SF to predict accurate SOC elements, making it particularly useful to complement the HAAM design concept. Furthermore and vital to instantiating the HAAM strategy, RAS-SF can produce natural transition orbitals (NTO) that couple pairs of spin states, revealing the specific changes in electronic structure that give rise to spin–orbit interactions. In this work, RAS-SF will show how the HAAM concept applies in practice to novel organic phosphors. This study therefore provides quantitative predictions of SOC and direct visualization of the interactions leading to SOC.

### 2.2. Implementation of HAAM strategy for molecular design

**Fig. 2:**
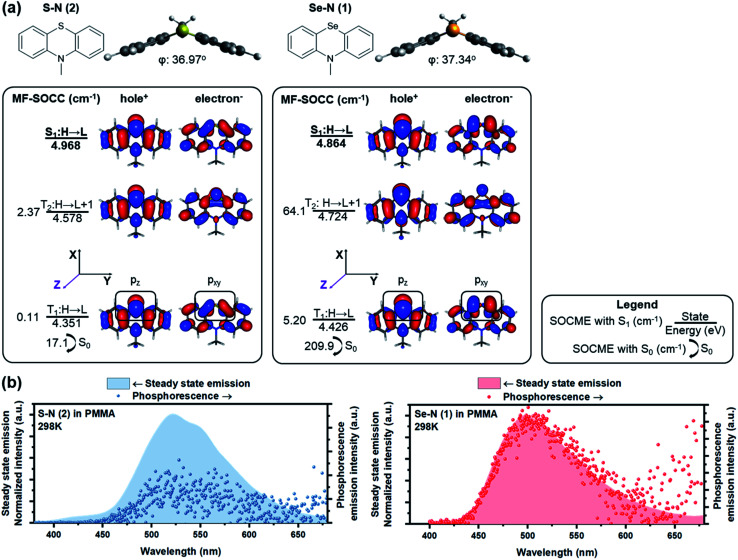
Computational and experimental results of Se–N (1) and Se–N (2). (a) Chemical structures of S–N and Se–N. Electronic structure results from RAS-SF showing the ground state optimized structure with bent geometry and dihedral angle and the NTOs of S_1_, T_1_, T_2_ states. Energy of each state and the selected mean field SOCMEs are shown as well. (b) Steady state emission spectra (filled graph, left axis) and gated phosphorescence emission spectra (dot, right axis, 0.5–5 ms for Se–N and 0.5–20 ms for S–N) of the two emitters doped in atactic PMMA (1 wt%, spin-cast) measured at room temperature in vacuum.

Increasing the involvement of heavy atoms in Δ*L* is critical to the HAAM concept, and the best amplification should be achieved where heavy atom orbitals are involved in the electronic transition of interest. Thus, the utilization of non-bonding electrons from heavy atoms is critically important. Chalcogen was selected as the heavy atom since, unlike halogens, it could be incorporated in the core of POPs to activate Δ*L* channels on its own, as shown in our previous work.^24^ To access the non-bonding p electrons from Se, we designed *N*-methylated phenylselenazine (Se–N, 1) carrying a bent geometry enabled by sp^3^ hybridization at the nitrogen (Fig. 2a). Accordingly, p electrons from Se are expected to be partially decoupled from the surrounding π-conjugated system and the non-bonding electrons could participate in Δ*L*.

RAS-SF calculations of Se–N showed a large SOCME of 209.9 cm^−1^ between T_1_ and the ground state S_0_, which is the critical transition affecting phosphorescence rate. A 64.1 cm^−1^ SOCME for the S_1_–T_2_ transition was also calculated, for the critical ISC process to populate triplet excitons. A detailed analysis on the NTOs of Se–N was performed to elucidate the high SOC efficiency of Se–N and its connections with the HAAM concept. As shown in Fig. 2a, p_*z*_ electrons of Se populated the hole orbital of T_1_ while p_*xy*_ (linear combination of p_*x*_ and p_*y*_, denoting orbitals in the *xy* plane in general) populated the electron orbital. Since orbital angular momentum rotates 90° between p_*z*_ and p_*xy*_ orbitals, electron migration between the hole and electron orbitals of T_1_ would carry a large “heavy atom oriented” Δ*L*, enabled by the heavy Se atom. In other words, the T_1_–S_0_ transition of Se–N follows the HAAM concept, leading to its large SOCME. Similarly, HAAM is also manifested in the S_1_–T_2_ SOC: the transition occurs through the excited electron orbitals of the S_1_ and T_2_ states, which are populated by p_*xy*_ and p_*z*_ electrons of Se, respectively.

To further test the novel design principle, Se–N was synthesized and embedded in polymeric matrixes (see methods for details†). In anoxic environment, fast room-temperature phosphorescence decay was observed (Fig. 2b), with measured *τ*_ph_ of 385 μs and *Φ*_ph_ of 19.2%. These results are consistent with the computed SOCME of 10^2^ cm^−1^, which is a record-high in POPs,^3^ and is comparable to that of some organometallic phosphors.^62–64^ This large SOCME enabled by the HAAM concept shows that POPs can have similar emissive properties compared to their organometallic counterparts, where SOCMEs are typically in the range of 10^2^–10^3^ cm^−1^.^62–64^

While the NTO analyses above provide qualitative visualization for how non-bonding p-electrons of Se directs Δ*L*, this phenomenon could also be quantified. Intuitively, if Δ*L* is dominated by a p_*z*_–p_*xy*_ transition, by using the right-hand rule, Δ*L* should be parallel to the *xy* plane. In other words, if we reduce the angular momentum change operator *L̂* using cartesian coordinates into *L̂*_*x*/*y*_ (*i.e. L̂*_*x*_ or *L̂*_*y*_) and *L̂*_*z*_, SOC efficiency will be more pronounced with the *L̂*_*x*/*y*_ operator, since the *L̂*_*z*_ operator only performs an orbital rotation around the *z* axis.

RAS-SF based NTO analyses not only produce accurate representation of SOC mechanism, but also provide insights on the reduced SOCME in selected orientations, which reveal the contributions of each angular momentum operator.^62^ In practice, according to the matrix representation of the angular momentum in the basis of p-orbitals, Se p_*z*_–p_*xy*_ transition in T_1_–S_0_ of Se–N should produce a considerable p_*z*_|*L̂*_*x*/*y*_|p_*xy*_ matrix element compared to that from the p_*z*_|*L̂*_*z*_|p_*xy*_ operator. This is confirmed by RAS-SF results (Table 1) showing major contributions to SOC from the reduced components in *L*_−_(*L*_*x*_) and *L*_+_(*L*_*y*_) orientations. Most importantly, the HAAM concept is directly supported since the majority of SOC is facilitated by Δ*L* on Se heavy atom. The benefit from RAS-SF methods is substantial in our discussion.

**Table 1:** Reduced SOCME in the selected orientations between S_0_ and T_1_ states

Orientation	S–N	Se–N
*L*_*x*_ or *L*_−_	−10.50 − 6.02*i*	−137.56 + 0.01*i*
*L*_*z*_ or *L*_0_	0.00	−78.77*i*
*L*_*y*_ or *L*_+_	−10.50 + 6.02*i*	−137.56 − 0.01*i*

The HAAM strategy was further examined by discretely tuning the heavy atom effect and Δ*L*. First, heavy atom effect was measured by replacing Se in Se–N with S while keeping Δ*L* relatively consistent. Frontier excited states of the designed molecule, S–N, have similar NTOs to those of Se–N, suggesting similar Δ*L* is present in the relevant electronic transitions. As expected, the SOCMEs of S–N were much smaller than those of the Se counterpart (*e.g.*, 17.1 *vs.* 209.9 cm^−1^ T_1_–S_0_ SOCME). Table 1 also indicates largely reduced SOCME of S–N for the reduced components in all three orientations.

**Fig. 3:**
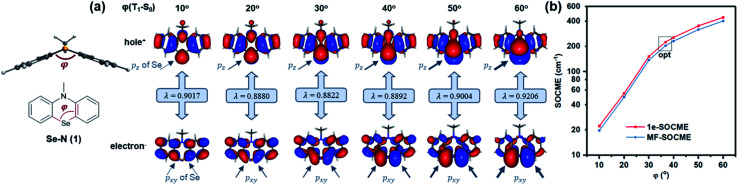
Computational results of Se–N (1) scanned through the dihedral angle. (a) T_1_–S_0_ NTOs of Se–N optimized with dihedral angle (*φ*) fixed; *λ* represents the contribution of the NTO pair in SOC (max. 1). (b) RAS-SF one-electron and mean-field SOCMEs of T_1_–S_0_ transition *vs.* dihedral angle, *φ*(°).

Secondly, Se was reintroduced and the contribution of heavy atom orbitals in Δ*L* was examined over a range of angular momentum changes. This was achieved *via* simulation by measuring the SOC for T_1_–S_0_ along various bending angles (Fig. 3a). The contribution of Se p_*xy*_ orbitals in the T_1_ state gradually increased as the bending angle was enlarged from 10 to 60° since p_*xy*_ electrons are gradually decoupled from the π-conjugated system, leading to the increased Δ*L* and T_1_–S_0_ SOCME (Fig. 3b). Similar trends also exist in the reduced SOCMEs in *L*_−_, *L*_0_, and *L*_+_ orientations (Fig. S1 and Table S2†).

**Fig. 4:**
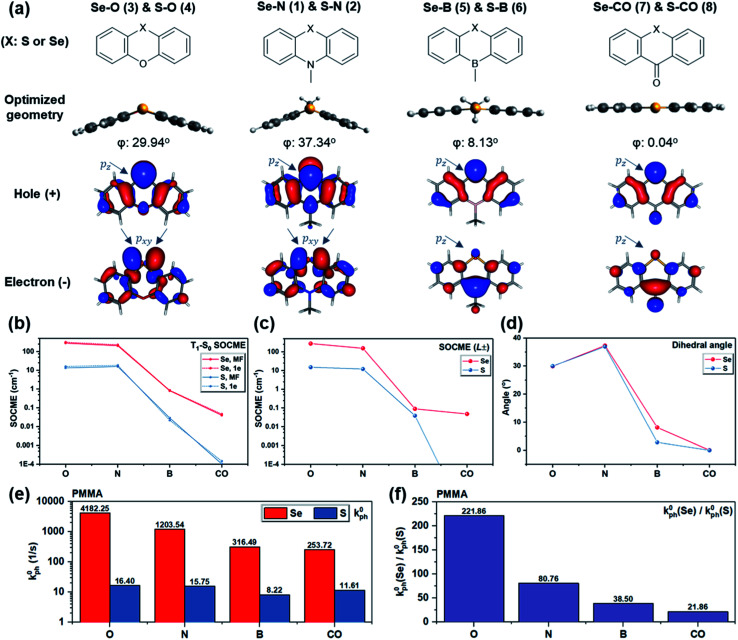
Computational and experimental results for S/Se–O, S/Se–N, S/Se–B, and S/Se–CO. (a) Molecular structures of the oxygen, nitrogen, boron, and carbonyl derivatives (X = S or Se), their optimized ground state geometry with dihedral angle marked, and RAS-SF NTOs of T_1_ states with Se p_*z*_ and p_*xy*_ orbitals marked; (b) RAS SF one-electron (1e) and mean-field (MF) SOCMEs of T_1_–S_0_ transition, (c) the reduced 1e SOCME in *L*_−_ or *L*_+_ orientations (modulus) *vs.* functional groups; (d) the dihedral angles of molecules studied *vs.* their functional groups; (e) the experimental intrinsic phosphorescence rate *k*^0^_ph_ measured in doped PMMA matrix at 78 K in vacuum, and (f) the *k*^0^_ph_(Se derivative)/*k*^0^_ph_(S derivative) value of each functional group.

To probe the dihedral degree of freedom *via* experiment, the functional group opposite to Se provides a possible handle. Thus, a series of molecules was designed where the nitrogen in Se–N is replaced by oxygen, boron, or carbonyl (Fig. 4a). With these substitutions, the dihedral angle was reduced from ∼37° to 0° (Fig. 4d) due to a change in orbital hybridization from sp^3^ (N) to sp^2^ (CO). Accordingly, Δ*L* is expected to decrease since non-bonding electrons of Se experience a higher degree of conjugation with the nearby ring system. Since Se is a much heavier atom than oxygen, boron, or carbonyl, the heavy atom effect should remain approximately constant. In line with expectations, the calculated T_1_–S_0_ SOCMEs show a decreasing trend upon reducing the bending angle from Se–N through Se–B to Se–CO (Fig. 4b and d). A similar trend is observed in the primary SOCME component in the *L*_±_ direction (Fig. 4c and Table S1†). Besides the bending angle, which affects the rotation of *L*, the induction effect of the substituents controls the electron density on Se and consequently the absolute value of *L*. This could explain the larger T_1_–S_0_ SOCME value of Se–O than that of Se–N, despite its smaller dihedral angle.

The computational results along with the molecular design rationale were experimentally checked by analyzing the intrinsic phosphorescence rate (*k*^0^_ph_) measured at 78 K. As expected, *k*^0^_ph_ decreased dramatically as the dihedral flattens in the series from Se–O to Se–CO (Fig. 4e). However, this observation alone only indirectly supports that the dihedral angle, or Δ*L* variation, leads to *k*^0^_ph_ variation. To gain further insight, *k*^0^_ph_ of each Se compound is compared with its S counterpart (*e.g.* Se–N *vs.* S–N). The experimental *k*^0^_ph_ of the S compounds doesn't experience a sharp decreasing trend from S–O to S–CO, but generally follows the prediction except for S–CO. We hypothesize that inevitable non-radiative decay for such long-lived triplet excitons in the sulfur compounds, as well as small mismatch between predicted and experimental dihedral angle could lead to unexpectedly larger *k*^0^_ph_ in the S compounds (see section V in ESI for details†).

Generally, while all four derivatives showed remarkable enhancement in *k*^0^_ph_ by replacing S with Se in the same molecular frame (Fig. 4e), the degree of *k*^0^_ph_ “boost”, characterized by *k*^0^_ph_(Se-derivative)/*k*^0^_ph_(S-derivative), varies with each molecular frame. It is worth addressing that the degree of *k*^0^_ph_ boost follows the dihedral angle, since a larger Δ*L* would enhance the contribution of heavy atom orbitals, and thus lead to more prominent heavy atom effects. Experimental results suggested the same trend (Fig. 4f) in the degree of *k*^0^_ph_ boost, which increased from Se–CO (*ca.* 22-fold), through Se–B, Se–N, to Se–O (a remarkable *ca.* 222-fold), strongly implying the beneficial effect of the increased dihedral angle and T_1_–S_0_ Δ*L*. These experimental results therefore demonstrate that the “heavy atom oriented” Δ*L* can be effective in practice, as motivated and expected by the HAAM concept.

## 3. Discussion

The analysis above implements the “heavy atom oriented orbital angular momentum manipulation” (HAAM) as a novel design feature for POPs and tested the concept using simulations and experiments. This HAAM strategy enables a powerful use of SOC theory in molecular design, and shows the potential to give control over SOC in triplet emitters. Highly efficient POPs with fast emission were realized with this strategy, having *k*^0^_ph_ over 10^3^ s^−1^ and promising room-temperature phosphorescence (RTP) properties.

This advance was supported by a novel computational method, RAS-SF, which can accurately quantify SOC in triplet emitters. RAS-SF also provides detailed NTO analysis to provide direct computational supports for the HAAM concept by revealing the electronic changes from triplet to singlet state that come with SOC. Importantly, the connection between experimental findings and systematic RAS-SF-assisted molecular design gave strong evidence that the tuning of heavy atom effects and orbital angular momentum—in synergy—is vital to increasing SOC in POPs. In other words, the combined quantitative and qualitative aspects of RAS-SF allowed the HAAM concept to be successfully translated from theory to practice for the design of POPs.

This combination of HAAM strategy with the RAS-SF SOC method can be extended to other organic triplet-based emitter scaffolds. Whereas the HAAM concept is enacted in practice to the Se series in this work, the key to efficient HAAM-based POPs is activating the contribution of heavy atom orbitals in Δ*L*, and the key to activate efficient Δ*L* channels is utilizing non-bonding electrons of heavy atoms. The “bent geometry” investigated here is expected to be just one efficient molecular scaffold to fulfill this task. A related, but less powerful method is to place heavy atoms adjacent to conventional El-Sayed rule satisfying moieties, so that they could participate in the (π,π*)−(n,π*) type Δ*L*. This design rationale employs halogen-containing POPs,^48,49^ since halogen only has one available bonding site and could not create efficient Δ*L* channels on their own.

In addition, while the fused-ring motif of the presented article appears to efficiently utilize the HAAM concept by connecting the heavy atom within the Δ*L*-producing channel, heavy chalcogen atoms could also be placed in attachable pendent groups to create Δ*L* channels, such as coupling them with ester or anhydride groups, or directly replace the oxygen in carbonyl with heavier chalcogens. However, these moieties haven't been systematically explored yet.

Further exploration of the HAAM strategy will focus on expanding the library of HAAM-based POPs in order to further break the SOC limits of POPs. Another promising direction is to create new OLEDs that outperform their metal-organic counterparts.

## Author contributions

W. S. synthesized the materials and conducted the photophysical analyses, and wrote the manuscript; H. J. performed all computation analyses and contributed to the computation sections of the article; R. A. provided the selenium precursor for the compound Se–N; J. K. and P. M. Z. supervised the research and revised this article.

## Conflicts of interest

The authors declare no competing financial interests. Further information and requests for the data that support the findings of this study should be directed to and will be fulfilled by the lead contact, Jinsang Kim (jinsang@umich.edu).

## Supplementary Material

SC-013-D1SC05689A-s001
