# Supplementary material for: Heavy atom oriented orbital angular momentum manipulation in metal-free organic phosphors
Source: Chem Sci. 2021 Dec 24;13(3):789–97. doi: 10.1039/d1sc05689a (PMC8768842; doi:10.1039/d1sc05689a)
Supplement: SC-013-D1SC05689A-s001 [file SC-013-D1SC05689A-s001.pdf]

Electronic Supplementary Material (ESI) for Chemical Science.
This journal is © The Royal Society of Chemistry 2021

Heavy Atom Oriented Orbital Angular Momentum Manipulation in Metal-Free Organic Phosphors

Wenhao Shao[†], Hanjie Jiang[†], Ramin Ansari[‡], Paul M. Zimmerman[*,†], Jinsang Kim[*,†,‡,§,○]

*[†]Department of Chemistry, [‡]Department of Chemical Engineering, [§]Department of Materials Science and Engineering, and [○]Macromolecular Science and Engineering, University of Michigan, Ann Arbor, Michigan, 48109, United States. *paulzim@umich.edu (P.M.Z.) *jinsang@umich,edu (J.K.)*

Contents

I. Additional experimental details ········ 2
II. Computational details ········ 8
III. Reduced SOCME in the selected orientations ········ 9
IV. Emission/excitation spectra ········ 11
V. Additional lifetime information ········ 19
VI. NMR spectra ········ 25
VII. References ········ 48

I. Additional experimental details

General

All chemicals used were purchased from Millipore Sigma or Fisher Scientific unless specified and used without further purification. (2-Bromophenyl)hydrazine was purchased from Oakwood Products, Inc.. Deuterated solvents for NMR spectroscopy (nuclear magnetic resonance) were purchased from Cambridge Isotope Laboratories. Phenoxathiine (S-O, 98.0+%) and 9H-thioxanthen-9-one (S-CO, 98.0+%) were purchased from TCI America and used without further purification. 9H-selenoxanthen-9-one (Se-CO) was purchased form Millipore Sigma and used without purification.

Physical measurements

Nuclear Magnetic Resonance (NMR) spectra were collected on Varian MR400 (400 MHz), Varian Vnmrs 500 (500 MHz), or Varian Vnmrs 700 (700 MHz) spectrometer as indicated. Photoluminescence spectra were collected on a Photon Technologies International (PTI) QuantaMaster spectrofluorometer (QM-400) equipped with an integrating sphere (K-Sphere) and a cryostat. The emitters were doped in atactic PMMA matrix for solid-state measurements: quartz substrates (1.5*2.5 cm) were prepared and cleaned by sonication consecutively in soap, deionized water, acetone, isopropyl alcohol, and then proceeded to UV-ozone treatment for 30 min. Chloroform solution containing 0.025 wt% emitter and 2.5 wt% PMMA was prepared and spin-coated on the cleaned quartz substrates (500 rpm for 5 min). Last, the films were transferred into a glovebox filled with N_2 and baked at 120 ^{o}C for 30 min.

Synthesis of prototype molecules

We've designed a series of molecules in this report to provide systematic experimental support for the HAAM concept, as well as to demonstrate the capability of the HAAM concept in creating highly efficient POPs. The synthetic scheme of 10-methyl-10*H*-phenothiazine (S-N) and 10-methyl-10*H*-phenoselenazine (Se-N) were adopted and modified from ref 1. The scheme of 10-mesityl-10*H*-dibenzo[*b,e*][1,4]thiaborinine (S-B) and 10-mesityl-10*H*-dibenzo[*b,e*][1,4]selenaborinine (Se-B) were adopted and modified from ref 2. The scheme of phenoxaselenine (Se-O) was adopted from ref 3. Phenoxathiine (S-O, 98.0+%) and 9H-thioxanthen-9-one (S-CO, 98.0+%) were purchased from TCI America and used without further purification. 9H-selenoxanthen-9-one (Se-CO) was purchased form Millipore Sigma and used without purification.

- **(Se-N, 1) 10-methyl-10H-phenoselenazine**

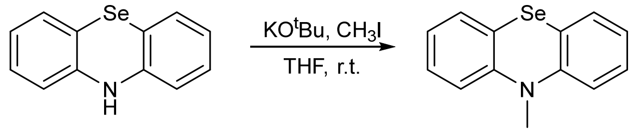

10*H*-phenoselenazine **Se-N (1)**

The synthetic procedure for 10*H*-phenoselenazine was described in ref 4.

10*H*-phenoselenazine (98 mg, 0.4 mmol, 1 eq.) and potassium tert-butoxide (67 mg, 0.6 mmol, 1.5 eq.) were dissolved in anhydrous THF (3 mL) at 0 °C. After stirring for 20 min, methyl iodide (142 mg, 1 mmol, 2.5 eq.) was added to the solution. The mixture was stirred at room temperature overnight. Then, the reaction was quenched with water and the resulting mixture was extracted with CH_2Cl_2. The combined organic layers were washed with brine,

dried over Na_2SO_4, filtered and evaporated to dryness under vacuum. The crude product was further purified with flash column chromatography (Hexane/CH_2Cl_2) to afford 10-methyl-10H-phenoselenazine (Se-N, **1**) as a white solid (17.0 mg, 16.4% yield).

- ^{1}H NMR (500 MHz, Chloroform-d) δ 7.32 (dd, J = 7.5, 1.4 Hz, 2H), 7.22 (td, J = 8.2, 1.4 Hz, 2H), 6.97 – 6.90 (m, 4H), 3.44 (s, 3H).
- ^{1}H NMR (500 MHz, DMSO-d6) δ 7.34 (dd, J = 7.5, 1.3 Hz, 2H), 7.29 – 7.23 (m, 2H), 7.03 (d, J = 8.1 Hz, 2H), 7.00 – 6.94 (m, 2H), 3.36 (s, 3H).
- ^{13}C NMR (126 MHz, DMSO-d6) δ 145.86, 129.89, 128.37, 123.40, 119.30, 116.20, 37.12.
- 77Se NMR (134 MHz, DMSO-d6) δ 278.89.

- **(S-N, 2) 10-methyl-10*H*-phenothiazine**

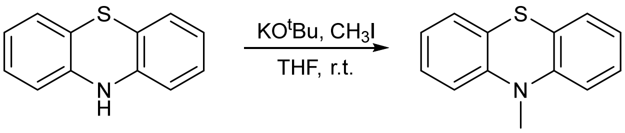

10*H*-phenothiazine

S-N (2)

10*H*-phenothiazine (1 g, 5 mmol, 1 eq.) and potassium tert-butoxide (0.845 g, 7.5 mmol, 1.5 eq.) were dissolved in anhydrous THF (10 mL) at 0 °C. After stirring for 20 min, methyl iodide (0.623 mL or 1.42 g, 10 mmol, 2 eq.) was added to the solution. The mixture was stirred at room temperature overnight. Then, the reaction was quenched with water, and the resulting mixture was extracted with CH_2Cl_2. The combined organic layers were washed with brine, dried over Na_2SO_4, filtered and evaporated to dryness under vacuum. The crude product was further purified with flash column chromatography (Hexane/CH_2Cl_2) to afford the pure 10-methyl-10*H*-phenothiazine (S-N, **2**) as a white solid (992.2 mg, 92.69% yield).

- 1H NMR (401 MHz, Chloroform-d) δ 7.21 – 7.10 (m, 4H), 6.98 – 6.88 (m, 2H), 6.82 (d, J = 8.1 Hz, 2H), 3.38 (s, 3H).
- 1H NMR (500 MHz, DMSO-d6) δ 7.24 – 7.19 (m, 2H), 7.15 (dd, J = 7.8, 1.0 Hz, 2H), 6.96 (t, J = 7.3 Hz, 4H), 3.30 (s, 3H).
- ^{13}C NMR (126 MHz, DMSO-d6) δ 145.78, 128.21, 127.22, 122.92, 122.53, 115.05, 35.56.

- **Dimethyl mesitylboronate, or MesB(OMe)$_2$**

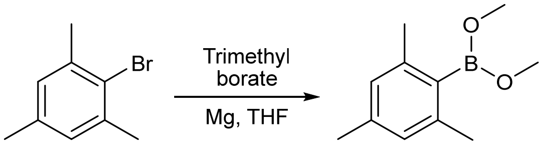

2-bromomestylene **MesB(OMe)$_2$**

(adopted and modified from ref 5,6.)

Mesityl magnesium bromide was prepared from 2-bromomesitylene (10.0 g, 50.23 mmol), magnesium turnings (1.343 g, 55.25 mmol, 1.1 eq.), and a small iodine crystal (127 mg, 0.5023 mmol, 0.01 eq.) in anhydrous THF (100 mL). After cloudiness appeared from the original clear solution, the mixture was refluxed for 2 hours at 80 ^{o}C or until all the magnesium disappeared, air-cooled to room temperature, and then slowly cooled to -78 ^{o}C using a liquid nitrogen/ethyl acetate bath. Trimethyl borate (6.440 mL, 57.76 mmol, 1.15 eq.) was added quickly and the solution was slowly warmed to room temperature overnight.

Afterwards, solvent was removed in vacuo and the residue was extracted with Ar-degassed hexanes. Solvent was completely removed from the crude mixture under reduced pressure to get an opaque oil which was distilled in vacuo (10^{-3} mbar, 130-200 °C) to obtain $MesB(OMe)_2$ as a colorless, transparent oil (4.069 g, 42.2% yield), which was stored in a desiccator in vacuum.

- ^{1}H NMR (400 MHz, Chloroform-d) δ 6.81 (s, 2H), 3.56 (s, 5H), 2.32 – 2.20 (m, 9H).
- ^{11}B NMR (128 MHz, Chloroform-d) δ 31.30.

▪ Bis(2-bromophenyl)selane

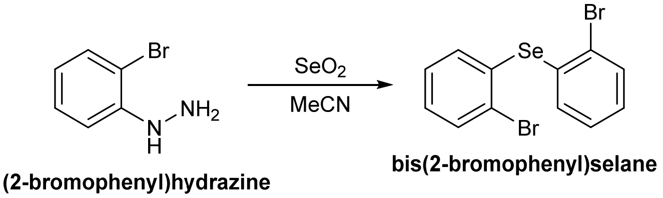

(adopted and modified from ref 7.)

2-bromophenyl)hydrazine (2 g, 10.69 mmol, 1 eq.) was dissolved in acetonitrile (25 mL) and added to a stirred suspension of selenium dioxide (1.78 g, 16.04 mmol, 1.5 eq.) in acetonitrile (25 mL). The suspension turned orange and then orange-red after bubble formation, and was then stirred at 60 °C for 1 h. Desired product was observed on TLC after 1 h and the resulting mixture was extracted with hexane to afford a red solution. After concentration under reduced pressure, the crude product was further purified with flash column chromatography (hexane) to afford bis(2-bromophenyl)selane as a yellow-orange solid (344.2 mg, 16.5% yield).

- 1H NMR (500 MHz, Chloroform-d) δ 7.63 (dd, J = 7.6, 1.6 Hz, 2H), 7.25 (dd, J = 7.6, 1.9 Hz, 2H), 7.23 – 7.13 (m, 4H).
- ^{13}C NMR (126 MHz, DMSO-d6) δ 134.44, 133.78, 132.71, 130.45, 129.44, 126.61.
- ^{77}Se NMR (95 MHz, Chloroform-d) δ 462.71.

▪ (Se-B, 5) 10-mesityl-10*H*-dibenzo[*b,e*][1,4]selenaborinine

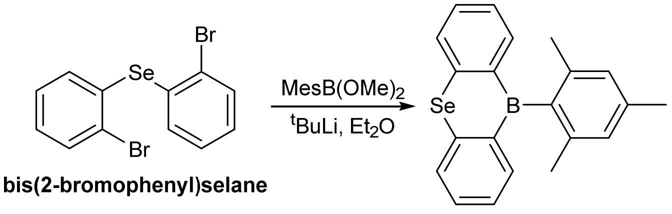

Se-B (5)

^{t}BuLi (1.9M pentane solution, 0.303 mL, 0.5766 mmol, 4.42 eq.) was added to a solution of bis(2-bromophenyl)selane (51 mg, 0.1304 mmol, 1 eq.) in diethyl ether (3 mL) at -78 °C. After stirring for 30 min at -78 °C, $MesB(OMe)_2$ (37.58 mg, 0.1957 μmol, 1.40 eq.) in diethyl ether (1.5 mL) was added to the reaction mixture. The mixture was warmed up to room temperature and stirred overnight before quenched with water. The organic layer was extracted with $CHCl_3$ and dried over anhydrous Na_2SO_4. After removal of the solvent under reduced pressure, the residue was purified with flash column chromatography (hexane) to afford 10-mesityl-10*H*-dibenzo[*b,e*][1,4]selenaborinine (Se-B, **5**) as a yellow solid (19.85 mg, 42.1% yield).

- ^{1}H NMR (500 MHz, Chloroform-d) δ 7.92 – 7.87 (m, 1H), 7.84 (d, J = 7.9 Hz, 1H), 7.57 – 7.51 (m, 1H), 7.29 (t, J = 7.4 Hz, 1H), 6.94 (s, 1H), 2.41 (s, 2H), 1.93 (s, 3H).
- ^{1}H NMR (401 MHz, DMSO-d6) δ 8.01 (d, J = 7.6 Hz, 1H), 7.72 (dd, J = 7.7, 1.3 Hz, 1H), 7.69 – 7.61 (m, 1H), 7.41 – 7.34 (m, 1H), 6.92 (s, 1H), 2.33 (s, 2H), 1.83 (s, 3H).
- ^{13}C NMR (126 MHz, DMSO-d6) δ 143.74, 140.39, 137.46, 136.11, 134.96, 132.75, 127.86, 127.07, 125.71, 118.06, 22.50, 20.85.
- ^{11}B NMR (128 MHz, Chloroform-d) δ 61.79.
- ^{77}Se NMR (95 MHz, Chloroform-d) δ 409.75.

Bis(2-bromophenyl)sulfane

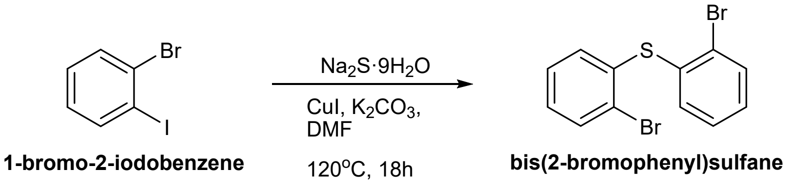

(adopted and modified from ref 8.)

CuI (38.5 mg, 0.202 mmol, 0.2 eq.), K_2CO_3 (279 mg, 2.02 mmol, 2 eq.), Na_2S (291 mg, 1.21 mmol, 1.2 eq.) and 1-bromo-2-iodobenzene (572 mg, 2.02 mmol, 2 eq.) were mixed in DMF (4 mL). The mixture was heated at 120°C overnight and allowed to cool to room temperature. The resulting mixture was extracted with ethyl acetate. The combined organic layers were dried over Na_2SO_4 and then concentrated under reduced pressure. The crude product was purified with flash column chromatography (hexane) to afford bis(2-bromophenyl)sulfane as white powder (126.4 mg, 36.3% yield).

- ^{1}H NMR (401 MHz, Chloroform-d) δ 7.65 (dd, J = 7.8, 1.2 Hz, 2H), 7.24 (dd, J = 7.6, 1.4 Hz, 2H), 7.18 – 7.11 (m, 4H).
- ^{1}H NMR (500 MHz, DMSO-d6) δ 7.79 – 7.75 (m, 2H), 7.42 – 7.38 (m, 2H), 7.30 (td, J = 7.6, 1.3 Hz, 2H), 7.13 (dd, J = 7.8, 1.4 Hz, 2H).
- ^{13}C NMR (126 MHz, DMSO-d6) δ 134.81, 134.02, 132.68, 130.13, 129.43, 125.35.

(S-B, 6) 10-mesityl-10*H*-dibenzo[*b*,*e*][1,4]thiaborinine

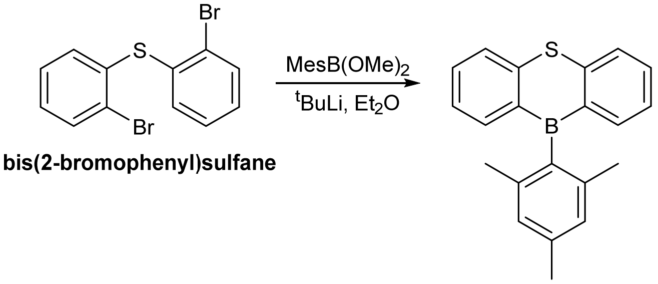

S-B (6)

$^{t}BuLi$ (1.9m pentane solution, 0.667 mL, 1.267mmol, 4.36 eq.) was added to a solution of bis(2-bromophenyl)sulfane (100mg, 0.2906 mmol, 1 eq.) in Et_2O (2 mL) at -78°C. After stirring for 30 min at -78°C, $MesB(OMe)_2$ (77mg, 0.4011mmol, 1.38 eq.) in Et_2O (3 mL) was added to the reaction mixture. The reaction mixture was stirred at r.t. overnight and the reaction was quenched with water.

The organic layer was extracted with $CHCl_3$ and dried over anhydrous Na_2SO_4. After removal of the solvent under reduced pressure, the residue was purified with flash column

chromatography (hexane) to afford 10-mesityl-10*H*-dibenzo[*b,e*][1,4]thiaborinine (S-B, **6**) as a pale yellow solid (45.67 mg, 43.51% yield).

- ^{1}H NMR (400 MHz, Chloroform-d) δ 7.88 (d, J = 7.7 Hz, 1H), 7.78 (d, J = 8.1 Hz, 1H), 7.62 (t, J = 7.5 Hz, 1H), 7.30 (t, J = 7.4 Hz, 1H), 6.95 (s, 1H), 2.41 (s, 2H), 1.93 (s, 3H).
- ^{1}H NMR (500 MHz, DMSO-d6) δ 7.94 (d, J = 8.1 Hz, 2H), 7.79 – 7.70 (m, 5H), 7.42 (t, J = 7.4 Hz, 2H), 6.94 (s, 2H), 2.34 (s, 3H), 1.83 (s, 6H).
- ^{13}C NMR (126 MHz, DMSO-d6) δ 143.90, 139.28, 138.10, 136.71, 133.42, 127.50, 126.04, 125.68, 23.00, 21.33.

- **1,2-bis(2-phenoxyphenyl)diselane**

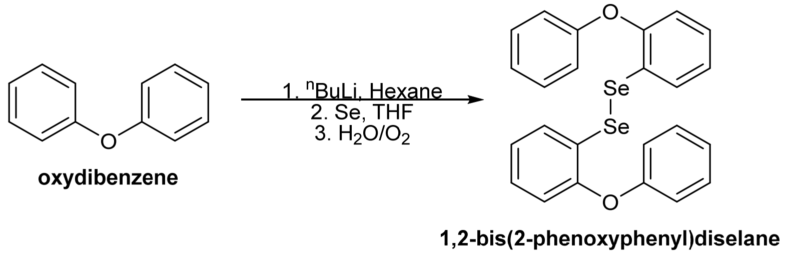

(adopted and modified from ref 3.)

1. To a solution of oxydibenzene (1.532 g, 9 mmol, 1eq.) in anhydrous hexane (25 mL), a 2.5 M solution of n-BuLi (9 mmol, 3.7 mL) in hexane was added dropwise at 0 °C and the reaction mixture was kept at this temperature for about 3 h with stirring. The reaction mixture was slowly warmed up to room temperature and it was left under stirring overnight.

2. The solvent was removed in vacuum and the remaining oil was dissolved in THF (20 mL). Afterwards, the stoichiometric amount of Se (710.64 mg, 9 mmol, 1eq.) was added and the solution became deep brown.

3. The reaction mixture was stirred for additional 2 h and then water was added (under argon) and subsequently the solution was exposed to air. The solvent was removed in vacuo and the remaining solid was dissolved in DCM (25 mL). The organic phase was washed with water and dried over anhydrous Na_2SO_4 to afford 1,2-bis(2-phenoxyphenyl)diselane as a red oil which turned into a solid after storage in freezer (2.076 g, 92.93% yield).

- ^{1}H NMR (700 MHz, Chloroform-d) δ 7.69 (d, J = 7.9 Hz, 2H), 7.34 (t, J = 7.5 Hz, 4H), 7.18 (t, J = 7.7 Hz, 2H), 7.12 (t, J = 7.4 Hz, 2H), 7.02 (dd, J = 21.5, 8.0 Hz, 6H), 6.83 (d, J = 8.1 Hz, 2H).
- ^{77}Se NMR (134 MHz, Chloroform-d) δ 354.66.

- **(Se-O, 3) Phenoxaselenine**

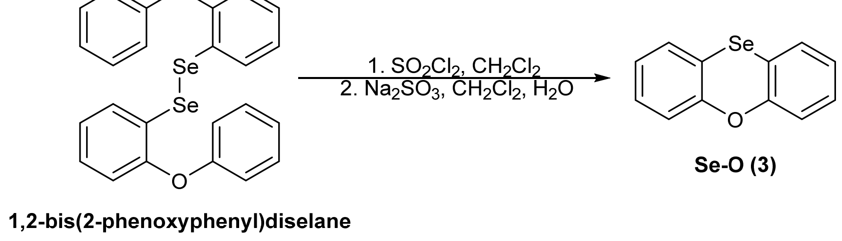

(adopted and modified from ref 3.)

1. To a solution of 1,2-bis(2-phenoxyphenyl)diselane (0.335 g, 0.675 mmol) in anhydrous DCM (10 mL), a 1 M solution of SO_2Cl_2 in CH_2Cl_2 (0.675 mL, 0.675 mmol) was added dropwise and the solution grew from light yellow to brown. The solution was left stirred for 2 days, and the color gradually changed back to yellow. After removal of all volatiles at reduced pressure, the remaining intermediate solid was washed with anhydrous hexane and dried.

2. To a solution of the intermediate in DCM (15 mL), Na_2SO_3 (0.0206 g, 0.16 mmol) in H_2O (15 mL) was added. The solution was stirred for 3 h. The organic phase was separated and dried over anhydrous Na_2SO_4, filtered, and concentrated at reduced pressure to afford phenoxaselenine (Se-O, **3**) as a white solid (135 mg, 31.5% yield).

- ^{1}H NMR (500 MHz, Chloroform-d) δ 7.30 (d, J = 7.6 Hz, 2H), 7.20 (t, J = 7.7 Hz, 2H), 7.12 (d, J = 8.0 Hz, 2H), 7.04 (t, J = 7.4 Hz, 2H).
- ^{1}H NMR (500 MHz, DMSO-d6) δ 7.47 (dd, J = 7.7, 1.4 Hz, 1H), 7.30 – 7.24 (m, 1H), 7.18 (dd, J = 8.1, 1.1 Hz, 1H), 7.12 (td, J = 7.5, 1.2 Hz, 1H).
- ^{13}C NMR (126 MHz, DMSO-d6) δ 152.68, 130.24, 129.11, 125.87, 118.96, 116.33.
- ^{77}Se NMR (134 MHz, Chloroform-d) δ 256.69.

II. Computational Details

The RAS-SF method is programmed in the Q-Chem 5.0 software package,[9] and the SOC computations are implemented in a development version of Q-Chem. All RAS-SF calculations were performed with the polarized, triple-zeta def2-TZVP basis set[10,11] and the RIMP2-cc-pVTZ auxiliary basis.[11] RAS-SF hole, particle calculations with 4 electron in 4 orbital active spaces were carried out with RAS1 and RAS3 subspaces including all occupied and virtual orbitals, respectively. Unless otherwise stated, the core electrons were kept frozen. Reference orbitals for RAS-SF were obtained from restricted open-shell density functional theory (RODFT) using the B3LYP functional in the triplet state. Geometries of the molecules were optimized at the ground state using ωB97X-D functional[12,13] and the def2-TZVP basis set[10,11]. Calculations of SOC constants utilize general libraries developed for SOC calculations within EOM-CC.[14] Spin-orbit NTOs were computed and analyzed using the libwfa library.[15] The NTOs with the largest singular values, for each compound, were plotted using Gabedit program.[16]

III. Reduced SOCME in the selected orientations

Table S1. Reduced SOCME in the selected orientations between S_0 and T_1 states

Orientation	L_x or L_-	L_z or L_0	L_y or L_+
S-O	14.8229+2.0238i	0.00	14.8229-2.0238i
Se-O	272.7789+45.2145i	0.00	272.7789-45.2145i
S-N	-10.50-6.02i	0.00	-10.50+6.02i
Se-N	-137.56+0.01i	-78.77i	-137.56-0.01i
S-B	-0.0390i	0.00	0.0399i
Se-B	-0.0002+0.0908i	0.00	-0.0002-0.0908i
S-CO	0.00	0.0001i	0.00
Se-CO	0.0001+0.0485i	0.00	0.0001-0.0485i

Table S2. Reduced SOCME in the selected orientations between S_0 and T_1 states, for Se-N with varying dihedral angle

Orientation	L_x or L_-	L_z or L_0	L_y or L_+
10°	18.2431+0.0069i	3.8320i	18.2431-0.0069i
20°	-37.2080+0.2711i	13.2820i	-37.2080-0.2711i
30°	-105.4036-0.0016i	-38.6745i	-105.4036+0.0016i
40°	173.2978-0.0001i	113.4250i	173.2978+0.0001i
50°	229.5948-0.0089i	223.7239i	229.5948+0.0089i
60°	-264.4984+0.0081i	-372.0783i	-264.4984-0.0081i

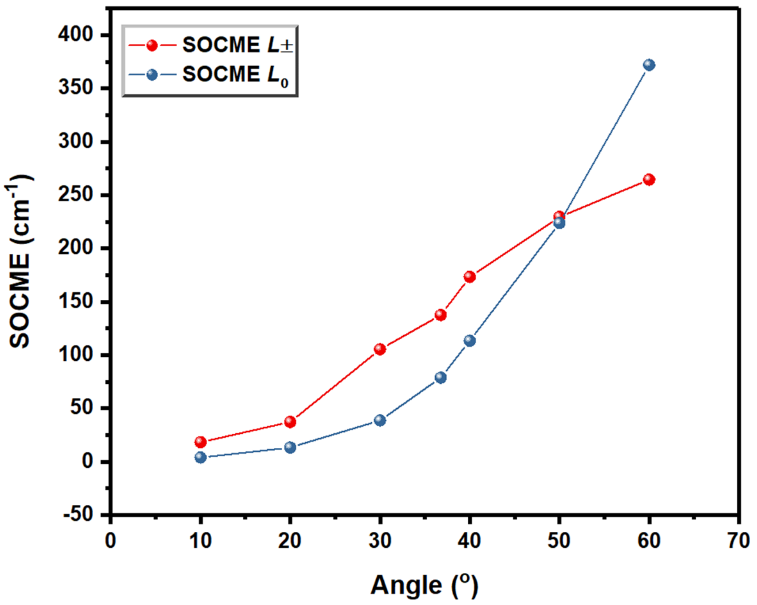

Figure S1. Reduced SOCME in the selected orientations between S_0 and T_1 states, for Se-N with varying dihedral angle (showing the modulus).

IV. Emission/excitation spectra

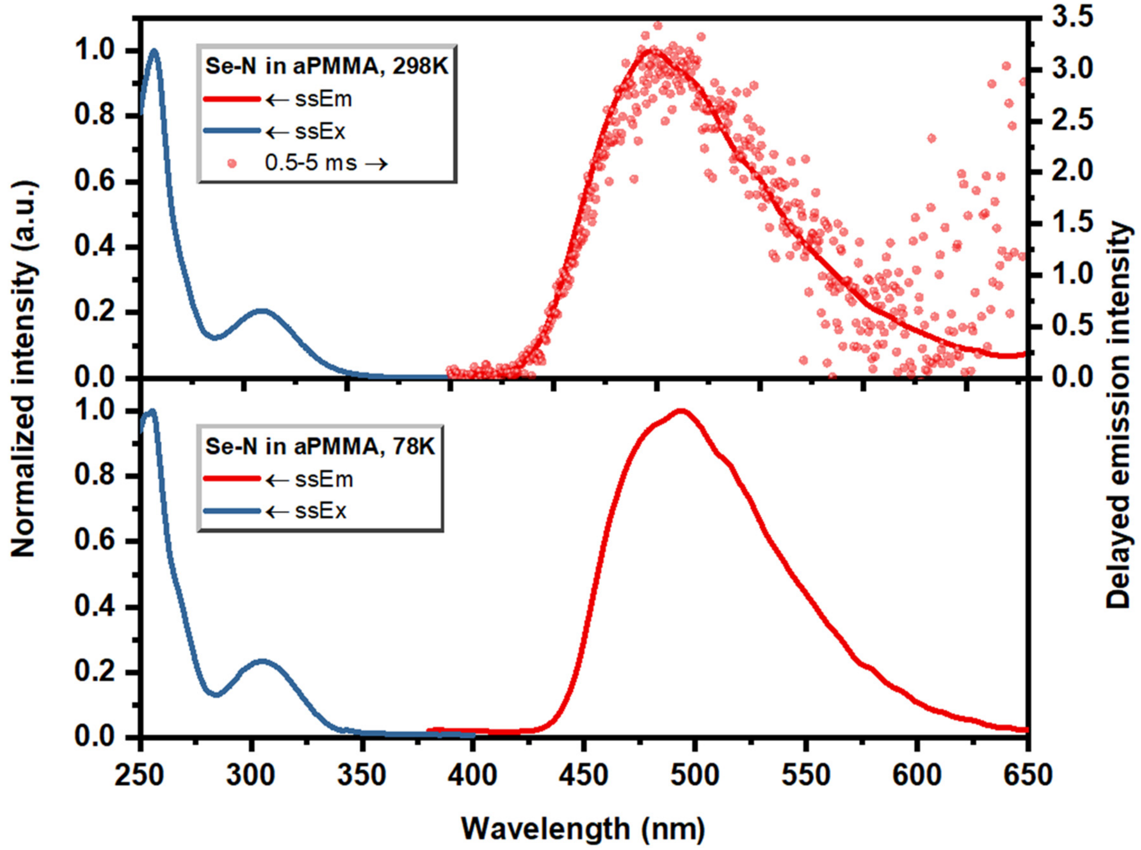

Figure S2. The emission and excitation spectra of Se-N (1 wt% in atactic PMMA matrix) at 298 K and 78 K measured in vacuum: steady state emission (red line), steady state excitation (blue line), and delayed emission (0.5-5 ms, red dot).

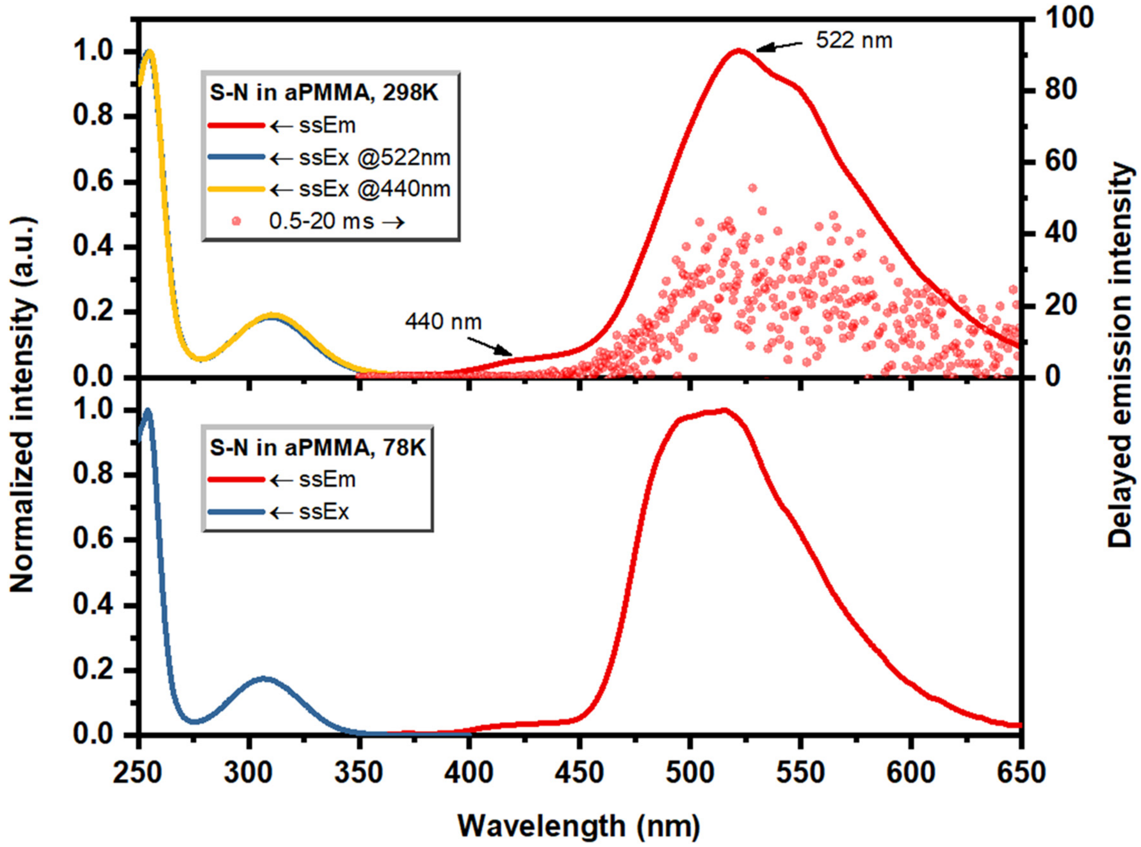

Figure S3. The emission and excitation spectra of S-N (1 wt% in atactic PMMA matrix) at 298 K and 78 K measured in vacuum: steady state emission (red line), steady state excitation (blue & yellow line), and delayed emission (0.5-5 ms, red dot).

The emission peak at 440 nm could either come from prompt fluorescence or delayed fluorescence.

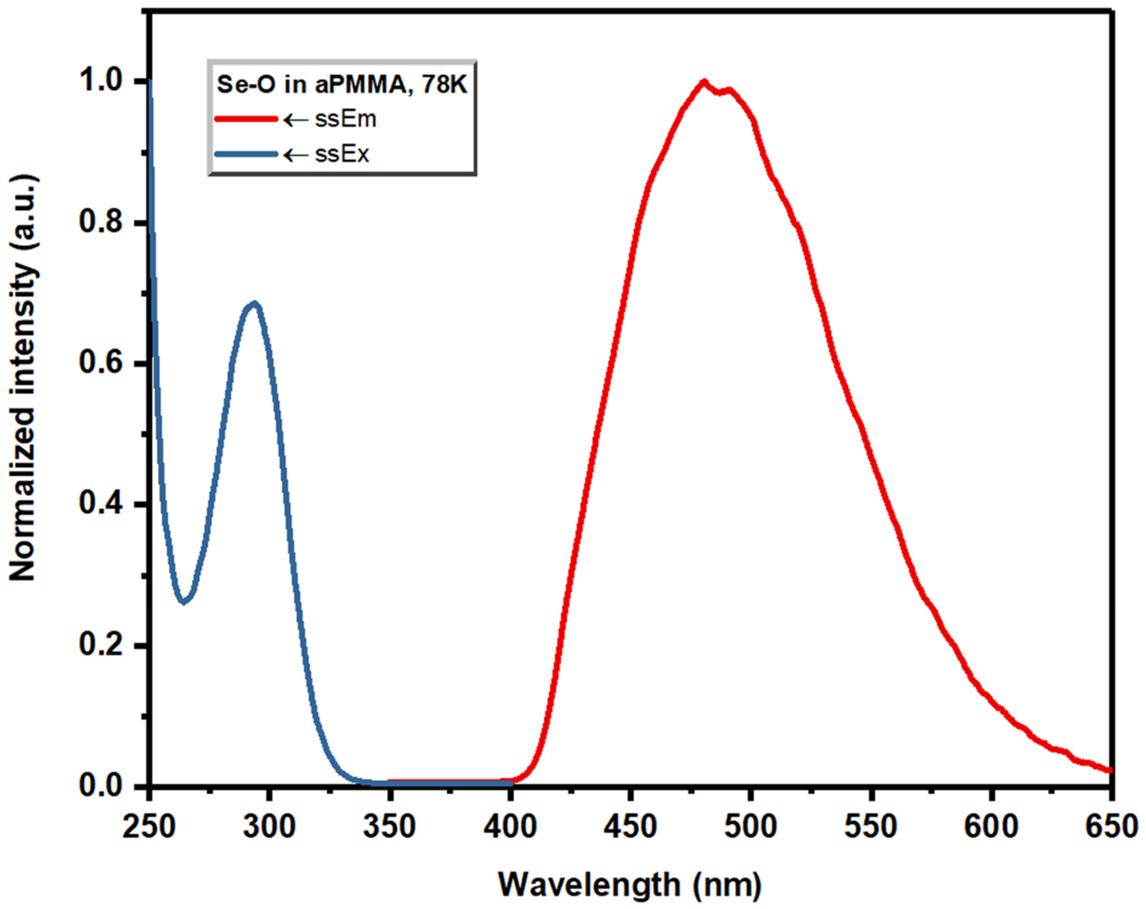

Figure S4. The emission and excitation spectra of Se-O (1 wt% in atactic PMMA matrix) at 78 K measured in vacuum: steady state emission (red line) and steady state excitation (blue line). The emission at 298 K was very weak, and thus it wasn't shown here.

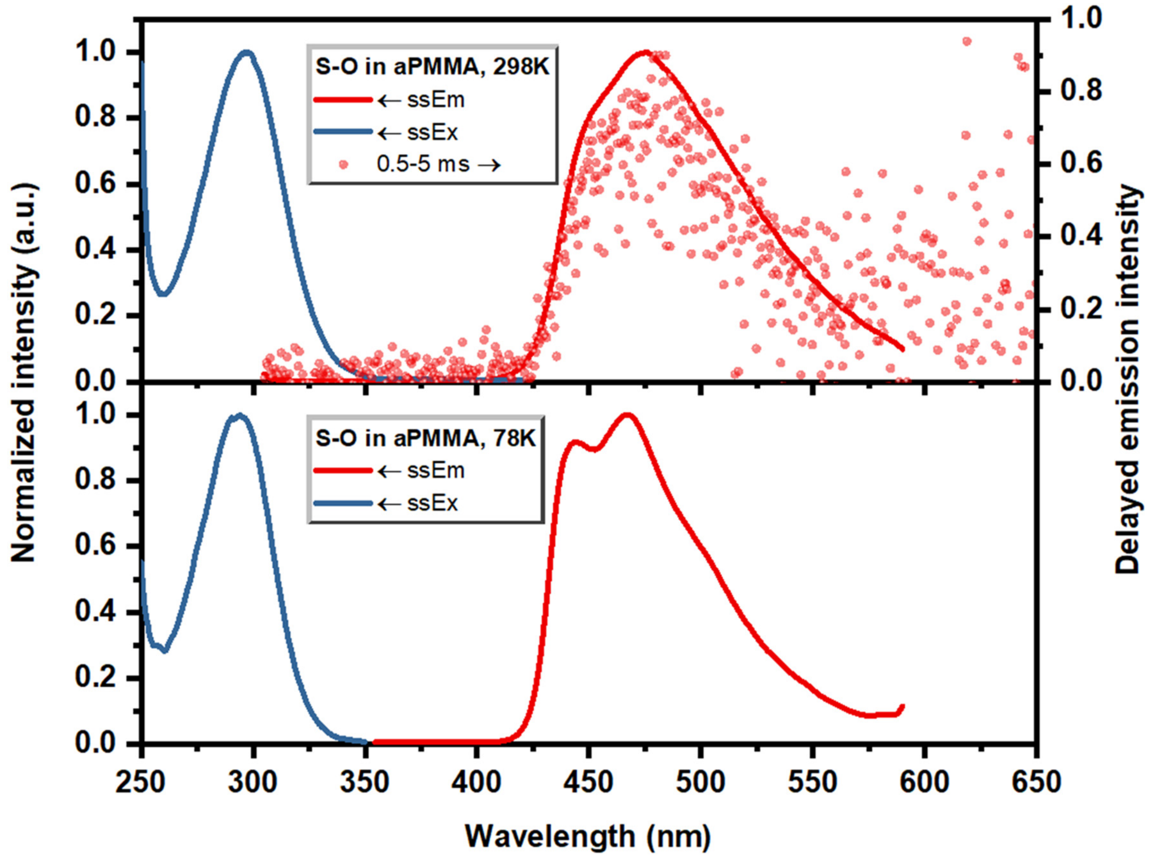

Figure S5. The emission and excitation spectra of S-O (1 wt% in atactic PMMA matrix) at 298 K and 78 K measured in vacuum: steady state emission (red line), steady state excitation (blue line), and delayed emission (0.5-5 ms, red dot).

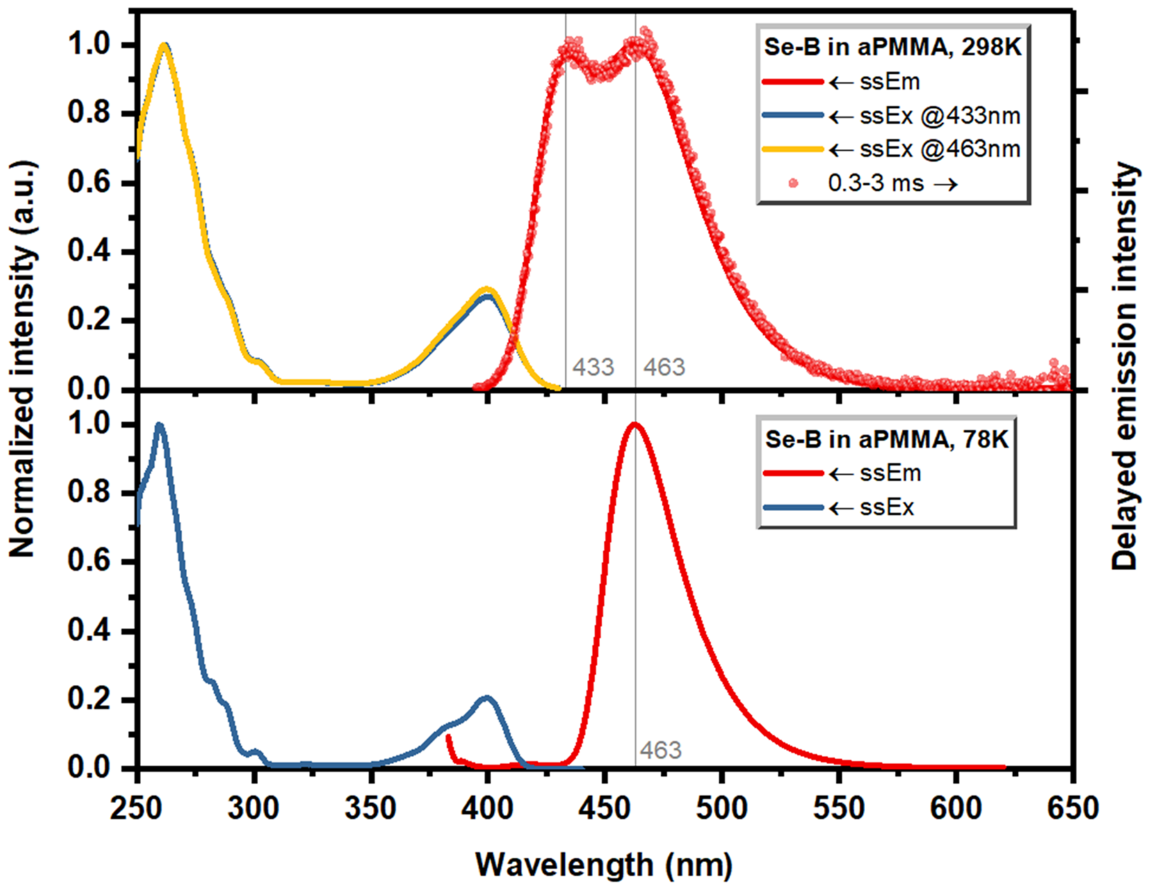

Figure S6. The emission and excitation spectra of Se-B (1 wt% in atactic PMMA matrix) at 298 K and 78 K measured in vacuum: steady state emission (red line), steady state excitation (blue & yellow line), and delayed emission (0.5-5 ms, red dot).

The emission peak at 433 nm (298 K) is from delayed fluorescence since it stayed the same relative intensity in the delayed spectrum, and disappeared at 78 K.

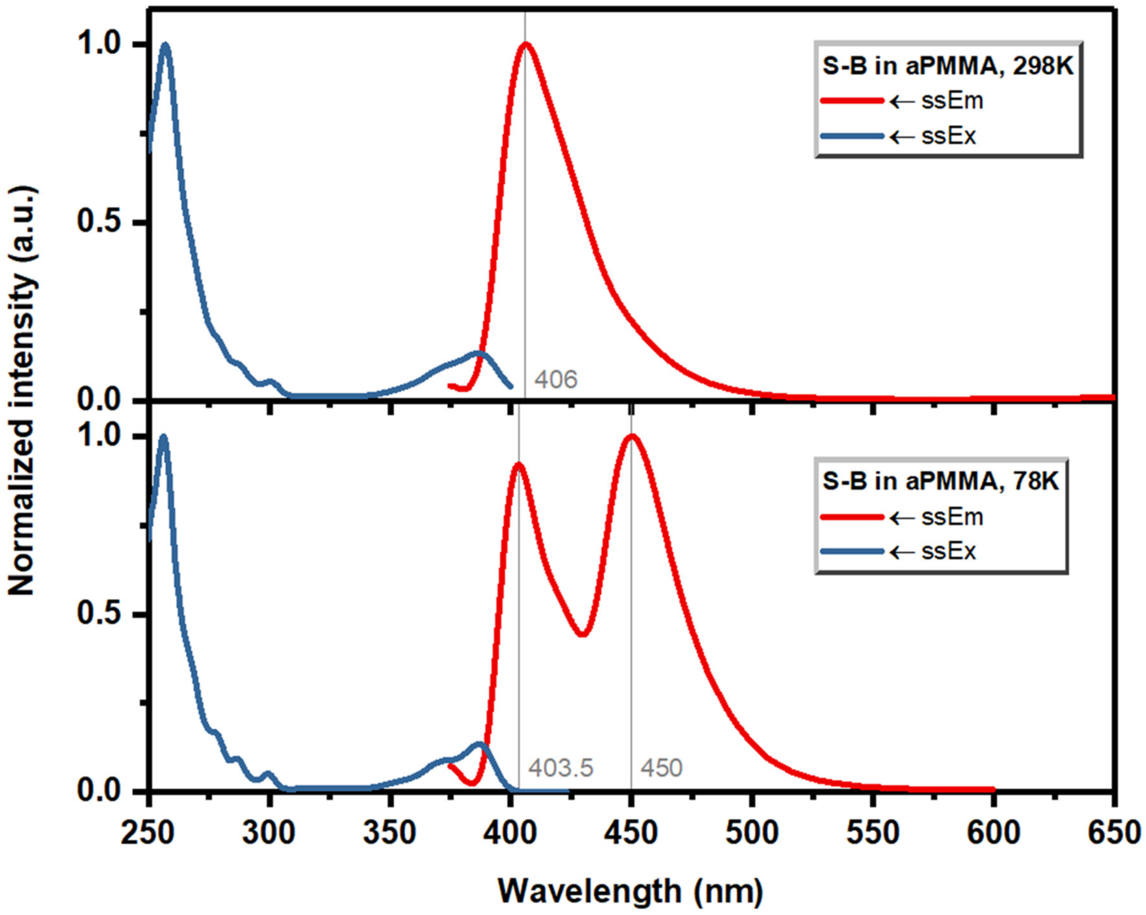

Figure S7. The emission and excitation spectra of S-B (1 wt% in atactic PMMA matrix) at 298 K and 78 K measured in vacuum: steady state emission (red line) and steady state excitation (blue line).

The emission peak at 406 nm (298 K) or 403.5 nm (78K) should have certain delayed fluorescence character.

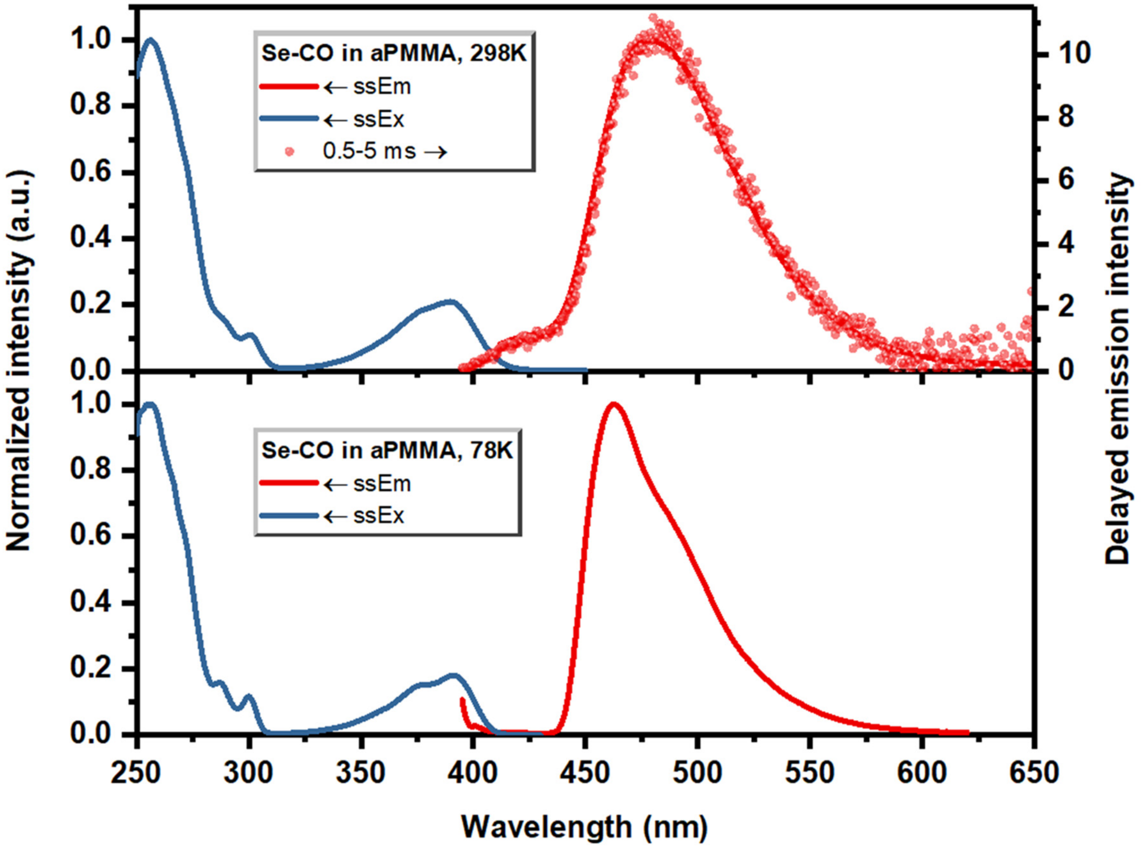

Figure S8. The emission and excitation spectra of Se-CO (1 wt% in atactic PMMA matrix) at 298 K and 78 K measured in vacuum: steady state emission (red line), steady state excitation (blue line), and delayed emission (0.5-5 ms, red dot).

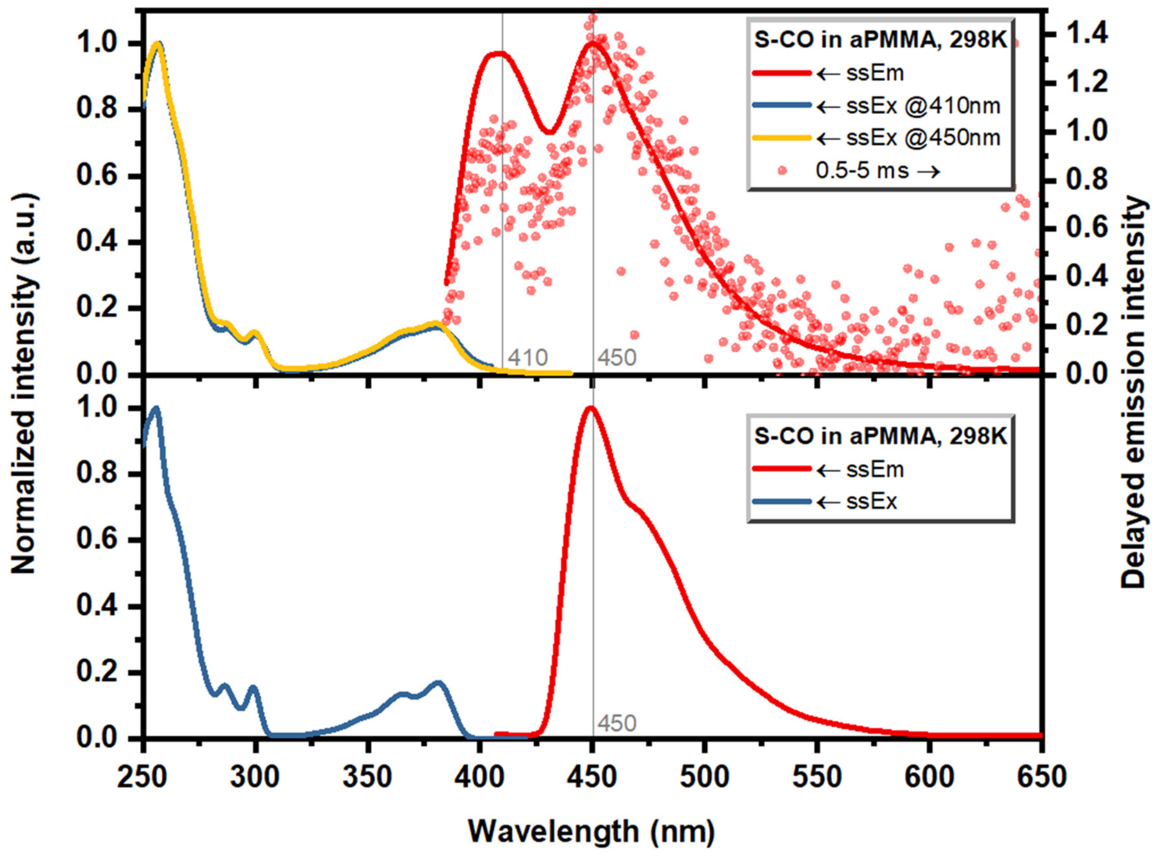

Figure S8. The emission and excitation spectra of Se-CO (1 wt% in atactic PMMA matrix) at 298 K and 78 K measured in vacuum: steady state emission (red line), steady state excitation (blue & yellow line), and delayed emission (0.5-5 ms, red dot).

The emission peak at 410 nm (298 K) should have certain delayed fluorescence character since it stayed the same relative intensity in the delayed spectrum, and disappeared at 78 K.

V. Additional lifetime information

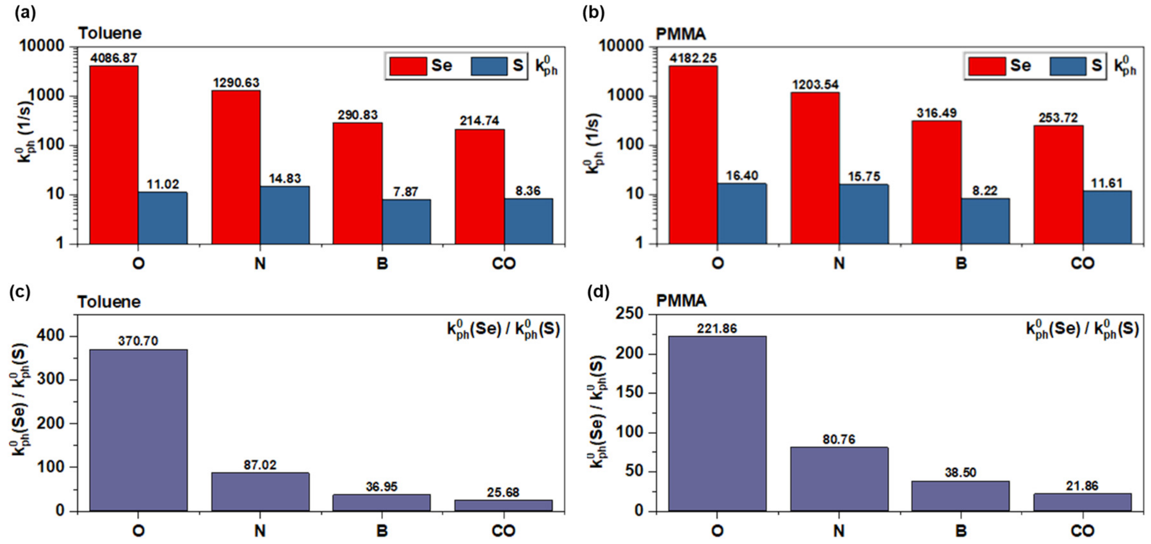

Figure S9. The experimental intrinsic phosphorescence rate k_{ph}^{0} measured in (a) toluene and (b) in doped PMMA matrix at 78 K, and the $k_{ph}^{0}(Se\ derivative)/k_{ph}^{0}\ (S\ derivative)$ value of each functional group in (c) toluene and (d) in doped PMMA matrix at 78 K.

The general trend where k_{ph}^{0} or SOCMEs decrease with decreasing dihedral angle is clearly observed in the Se compounds. In the sulfur series, the general trend in k_{ph}^{0} still follows prediction in that a sharp drop in dihedral angle from Se-O/N to Se-B/CO has led to obvious reduction in k_{ph}^{0}. This leaves just one case, the k_{ph}^{0} of S-CO, which is higher than that of S-B and therefore does not fit the trend.

We presume two plausible reasons. First, sulfur is not a heavy atom and due to the intrinsically lower SOCME, S derivatives will be more prone to non-radiative decay than their Se counterparts. This deactivation pathway comes from both the compound itself and its interaction with the matrices. Thus, the varying non-radiative decay rate from S-O, S-N to S-B and S-CO could lead to the deviation from the predicted T_1-S_0 SOCME trend.

Second, experimental dihedral angles of the two CO compounds are likely shifted from their calculated value. This is very important particularly for planar molecules since a little distortion from complete planarity could lead to large gain in T1-S0 SOCME. For instance, the predicted T_1-S_0 SOCME for Se-CO is 0.046 cm^{-1} and 1.04e^{-4} cm^{-1} for S-CO, which should both lead to very long intrinsic phosphorescence lifetime or very low k_{ph}^{0}. However, the experimental results in PMMA at 78K suggested otherwise. For instance, Se-CO has a fast-than-predicted τ_{ph}^{0} of 4.09 ms. Thus, we hypothesized that the experimental dihedral angle or more precisely, $\Delta \boldsymbol{L}$ in the CO series is non-negligible. Hence, the actual phosphorescence rate should be larger than the predicted value. To demonstrate these in experiments, we've compared the lifetimes of 8 compounds studied in dilute toluene solution with those in PMMA at 78K.

According to Figure S9, changing the matrix has inevitably changed the lifetime at 78K. This change is extremely large for S-CO which had k_{ph}^{78K} of 8.36 1/s in toluene v.s. 11.61 1/s in PMMA. S-CO and S-B now have similar lifetimes in toluene. We suspect that either non-radiative decay or dihedral angle change both due to interaction with the matrix may have led to the great lifetime change. Due to similar reasons, surprisingly, S-O presented similar large k_{ph}^{78K} change from PMMA to toluene. However, the overall lifetime trend is very obvious as predicted in the Se compounds, and hence the decreasing k_{ph}^{0} (Se derivative)/ k_{ph}^{0} (S derivative) as shown in Figure S9c-d. It is less pronounced in the S compounds, but follows the general reducing trend from S-N/O to S-B/CO.

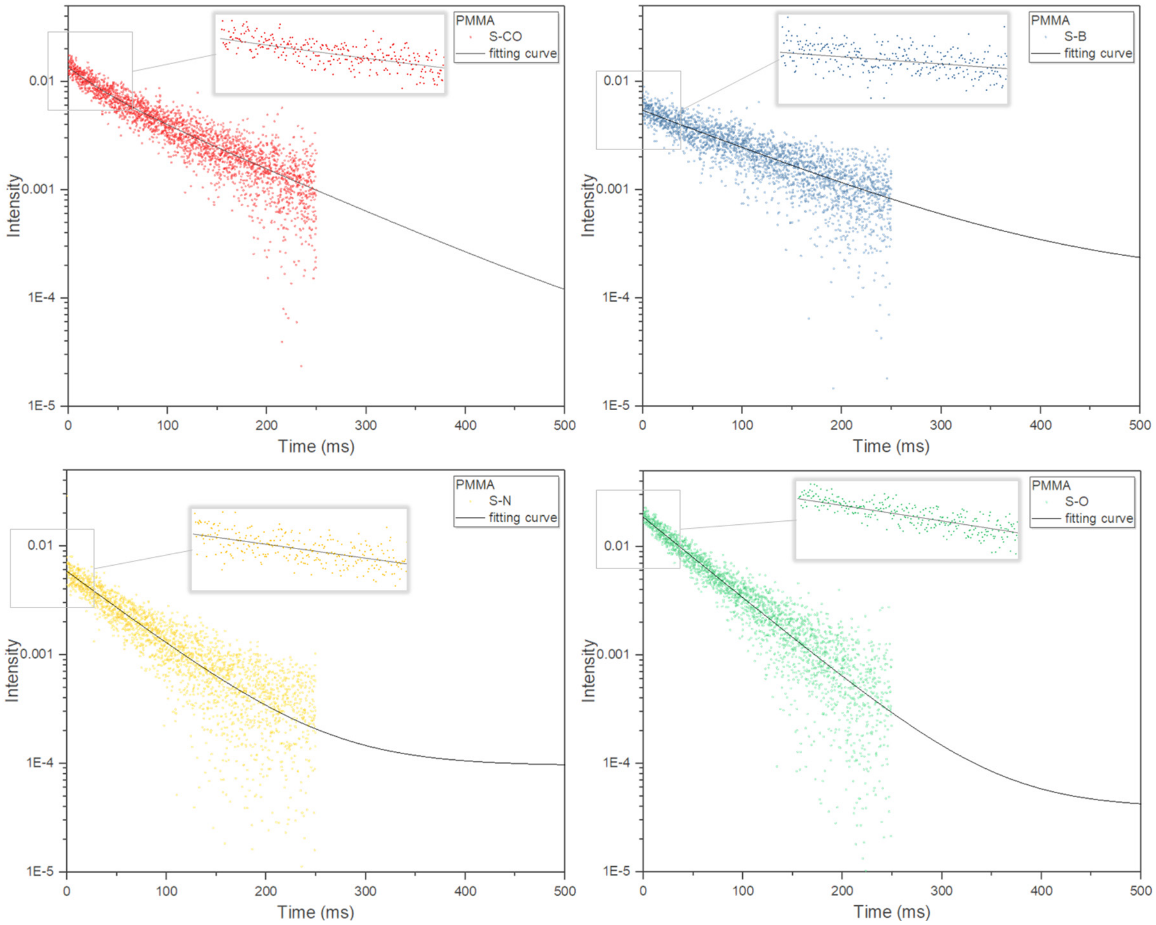

Figure S10. Photoluminescence decay of S-CO, S-B, S-N, and S-O in PMMA matrix measured at 78K. The fitting curve was included, which was done by Origin Lab software with its built-in exponential decay function. The raw decay data was recorded from 0 to 250 ms, while fitting was done using the data from 10 to 250 ms. The insets show the fitting in the head (1-30 ms). The head, body, and tail of the decay were all well-fitted, indicating good fitting quality.

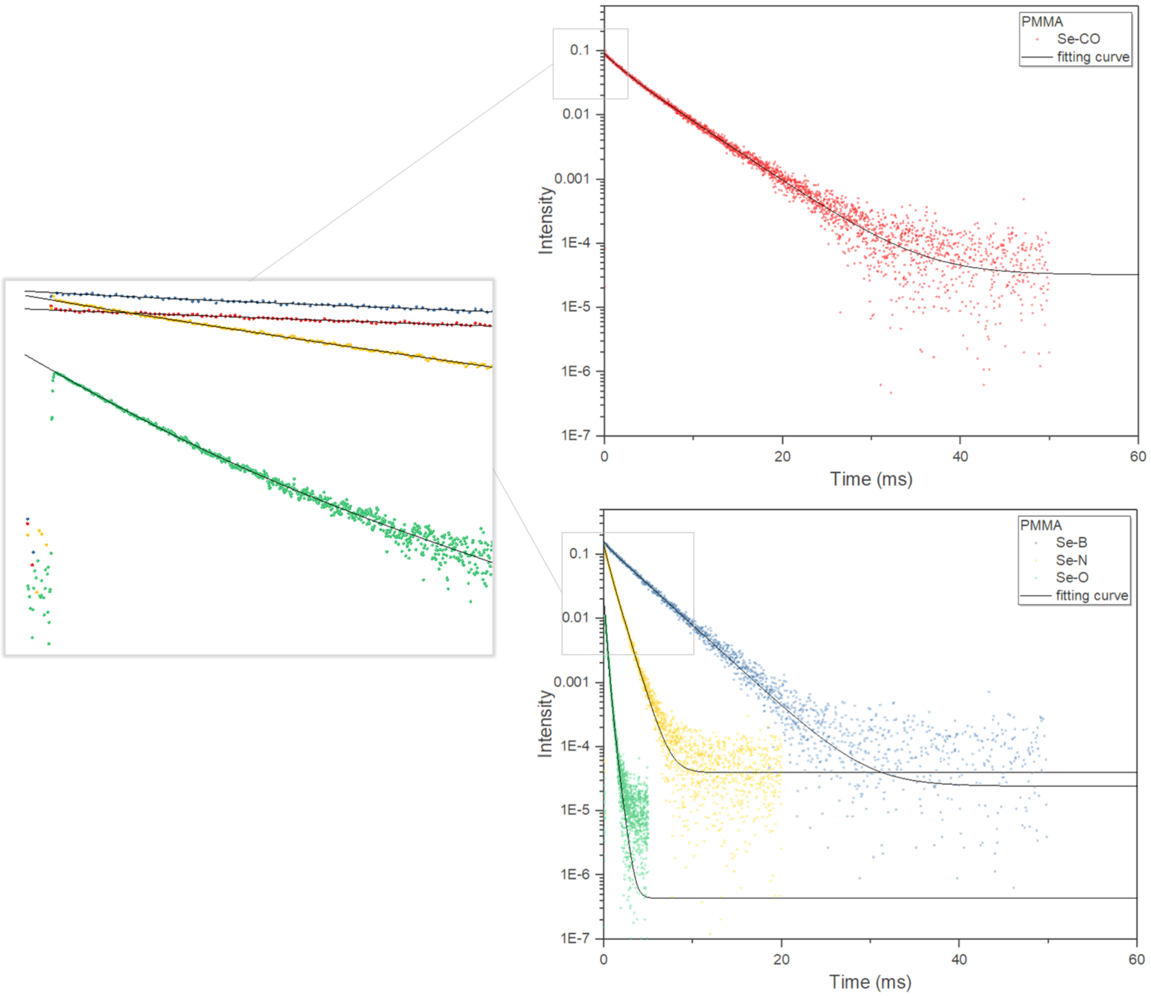

Figure S11. Photoluminescence decay of Se-CO, Se-B, Se-N, and Se-O in PMMA matrix measured at 78K. The fitting curve was included, which was done by Origin Lab software with its built-in exponential decay function. The raw decay data was recorded from 0 ms, while fitting was done using the data from 0.2 ms. The insets show the fitting in the head (0-2 ms). The head, body, and tail of the decay were all well-fitted, indicating good fitting quality.

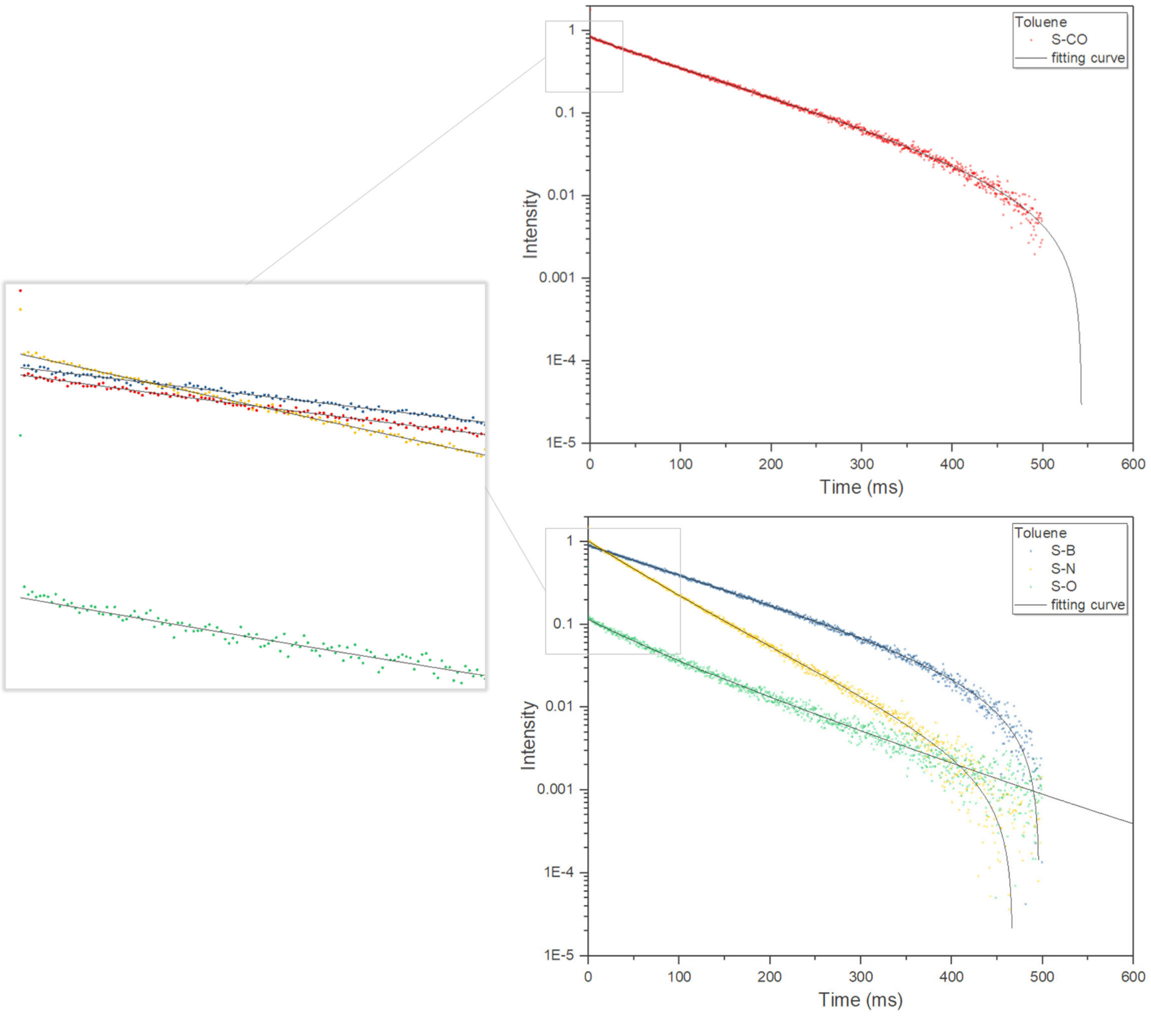

Figure S12. Photoluminescence decay of S-CO, S-B, S-N, and S-O in toluene measured at 78K. The fitting curve was included, which was done by Origin Lab software with its built-in exponential decay function. The raw decay data was recorded from 0-500 ms, while fitting was done using the data from 10 ms. The insets show the fitting in the head (0-60 ms). The head, body, and tail of the decay were all well-fitted, indicating good fitting quality.

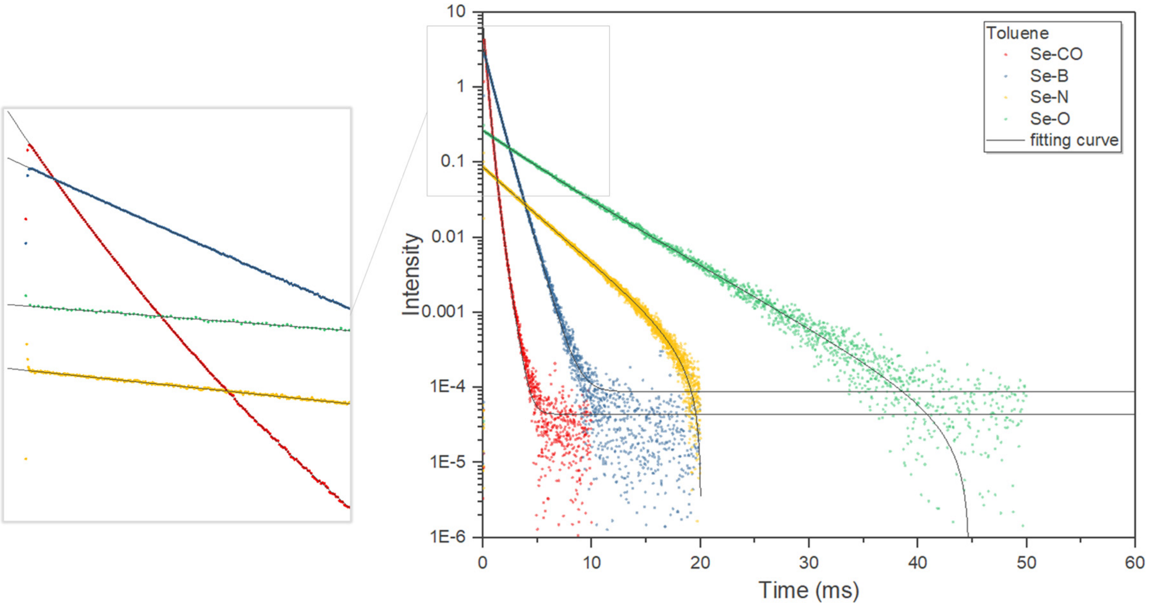

Figure S13. Photoluminescence decay of Se-CO, Se-B, Se-N, and Se-O in toluene measured at 78K. The fitting curve was included, which was done by Origin Lab software with its built-in exponential decay function. The raw decay data was recorded from 0 ms, while fitting was done using the data from 0.2 ms. The insets show the fitting in the head (0-2 ms). The head, body, and tail of the decay were all well-fitted, indicating good fitting quality.

VI. NMR spectra

- **(Se-N, 1) 10-methyl-10H-phenoselenazine**

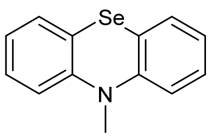

<u>^{1}H NMR (500 MHz, Chloroform-d)</u>

δ 7.32 (dd, J = 7.5, 1.4 Hz, 2H), 7.22 (td, J = 8.2, 1.4 Hz, 2H), 6.97 – 6.90 (m, 4H), 3.44 (s, 3H).

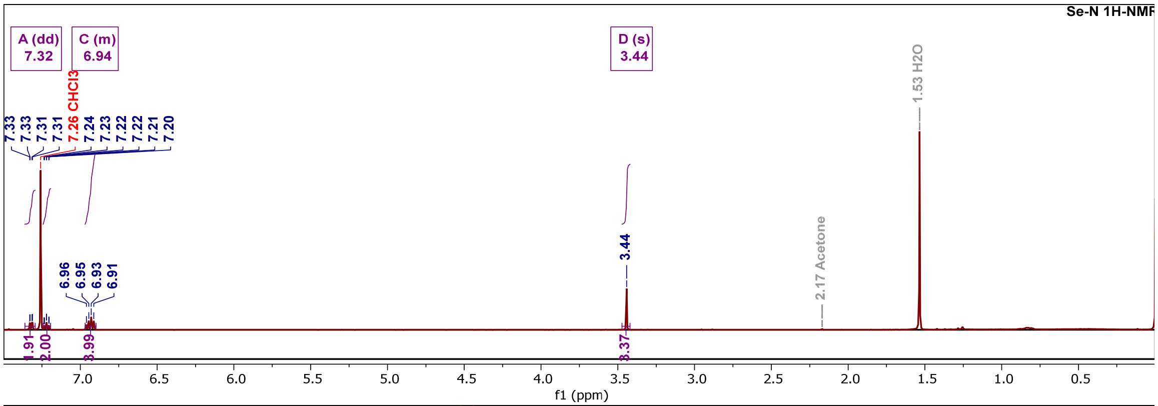

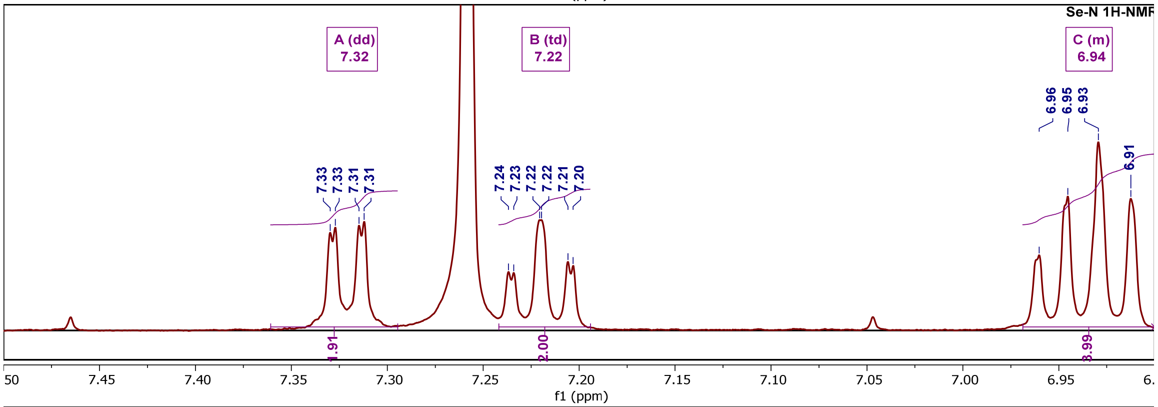

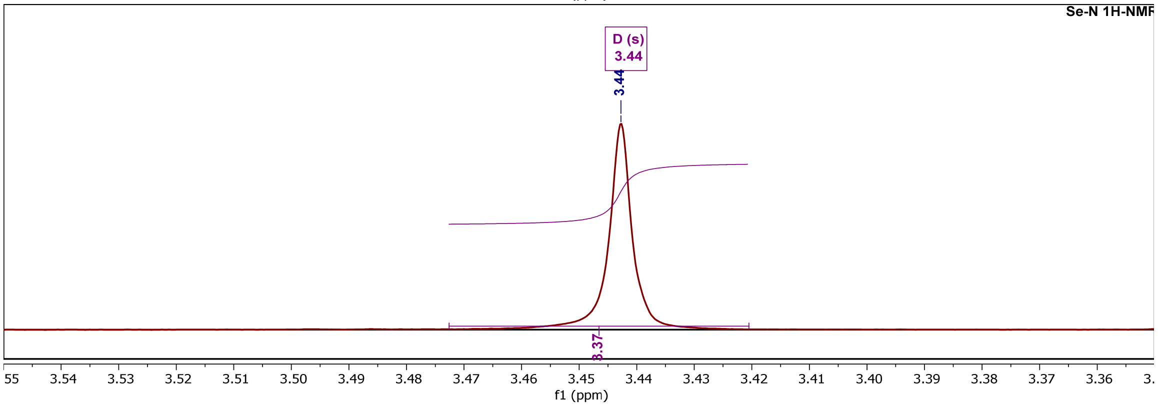

- **(Se-N, 1) 10-methyl-10H-phenoselenazine**

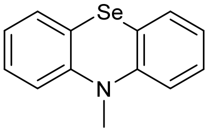

^{1}H NMR (500 MHz, DMSO-d6)

δ 7.34 (dd, J = 7.5, 1.3 Hz, 2H), 7.29 – 7.23 (m, 2H), 7.03 (d, J = 8.1 Hz, 2H), 7.00 – 6.94 (m, 2H), 3.36 (s, 3H).

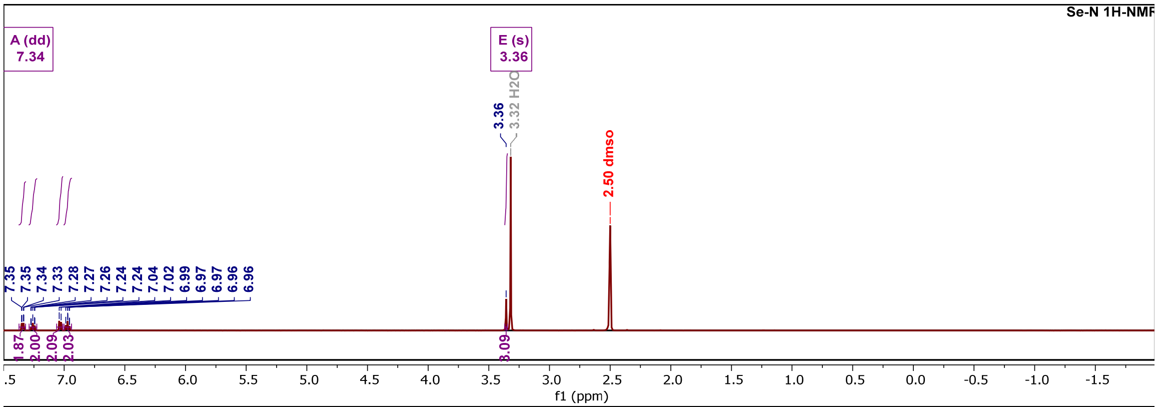

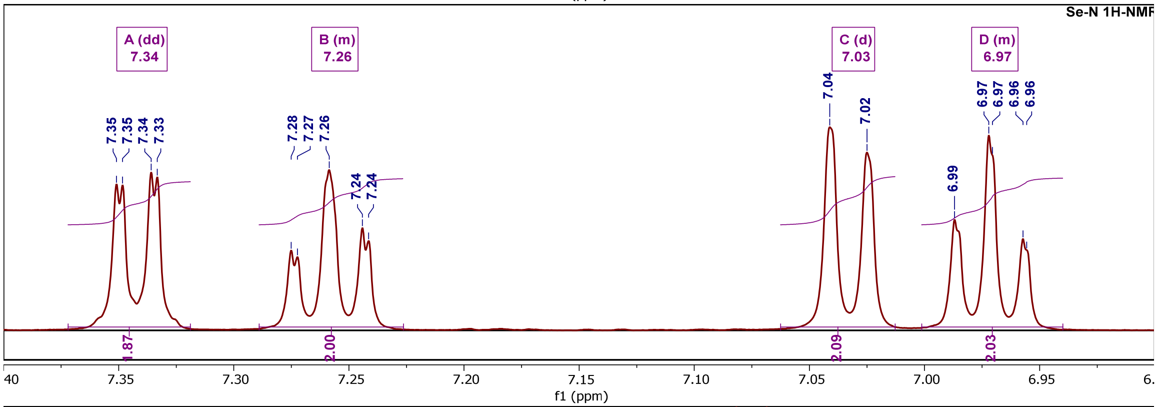

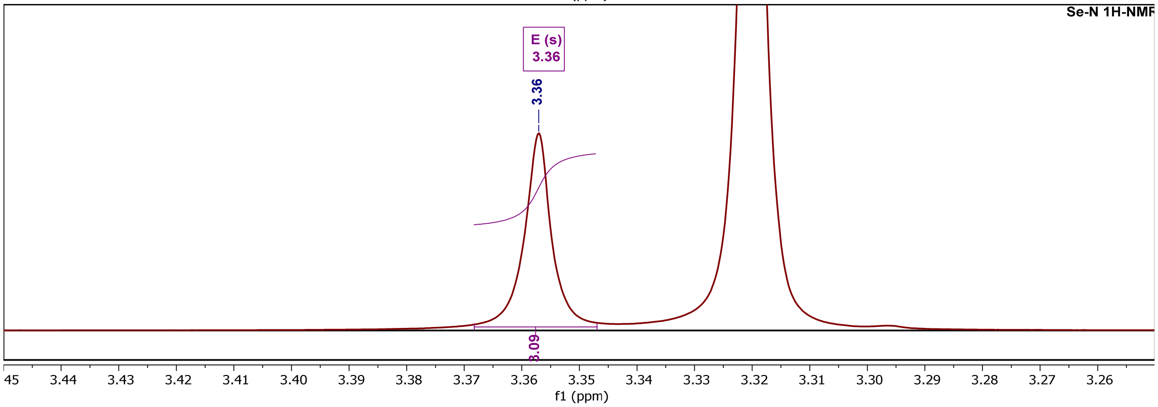

- **(Se-N, 1) 10-methyl-10H-phenoselenazine**

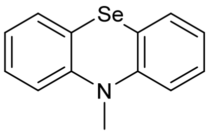

^{13}C NMR (126 MHz, DMSO-d6)

δ 145.86, 129.89, 128.37, 123.40, 119.30, 116.20, 37.12.

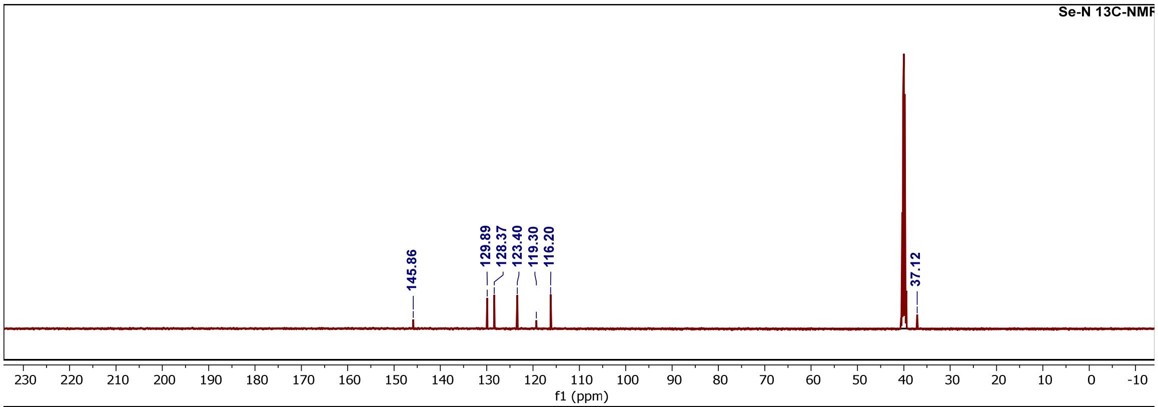

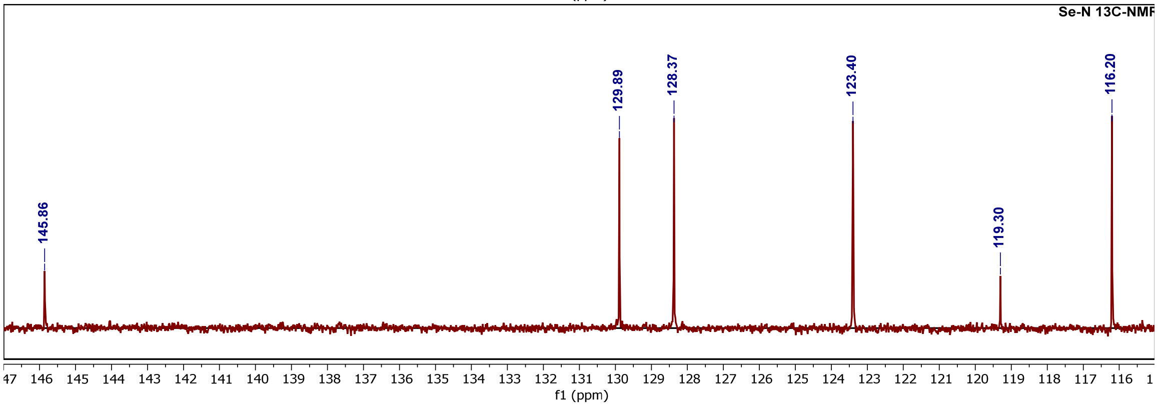

77Se NMR (134 MHz, DMSO-d6)

δ 278.89.

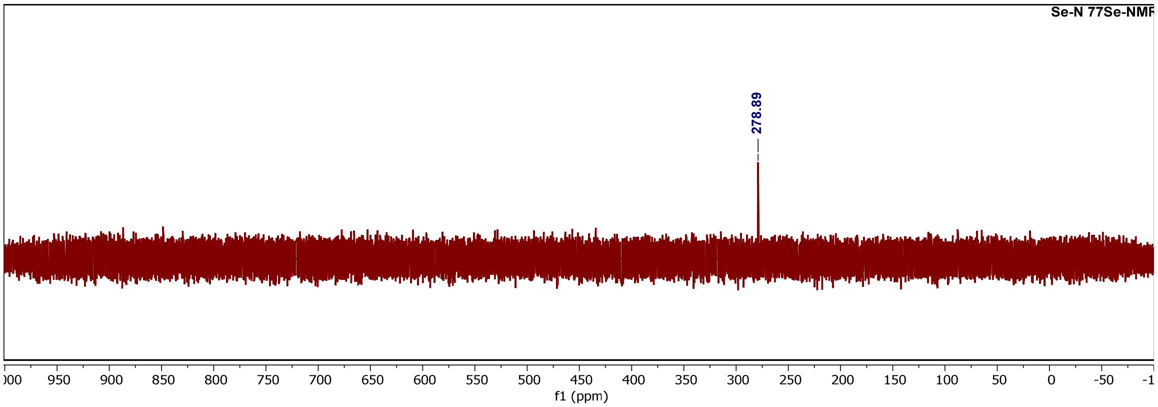

▪ **(*S*-N, 2) 10-methyl-10*H*-phenothiazine**

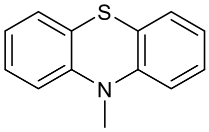

<u>*1H NMR (401 MHz, Chloroform-d)*</u>

δ 7.21 – 7.10 (m, 4H), 6.98 – 6.88 (m, 2H), 6.82 (d, J = 8.1 Hz, 2H), 3.38 (s, 3H).

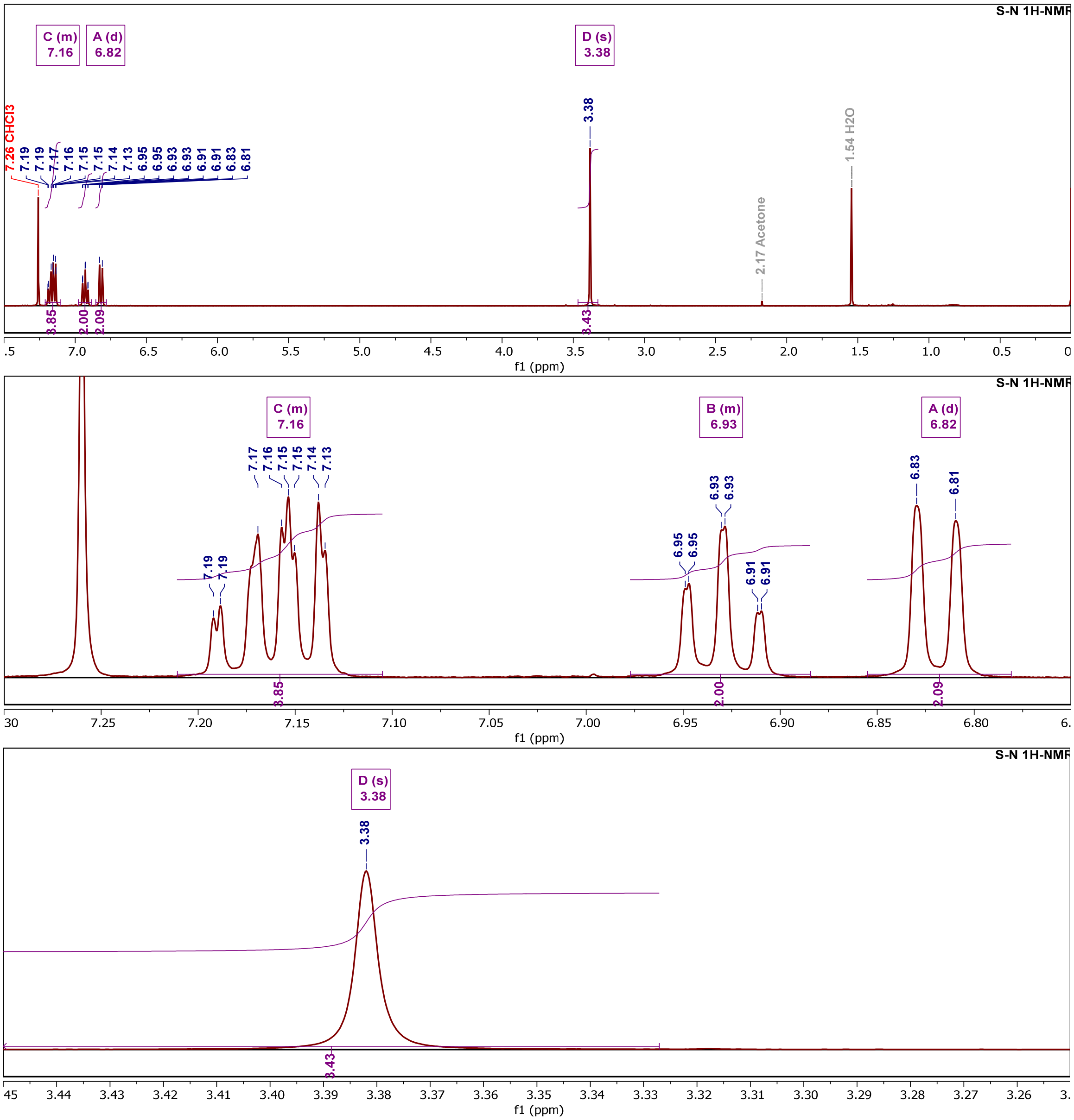

- **(*S*-N, 2) 10-methyl-10*H*-phenothiazine**

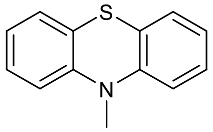

<u>*1H NMR (500 MHz, DMSO-d6)*</u>

δ 7.24 – 7.19 (m, 2H), 7.15 (dd, J = 7.8, 1.0 Hz, 2H), 6.96 (t, J = 7.3 Hz, 4H), 3.30 (s, 3H).

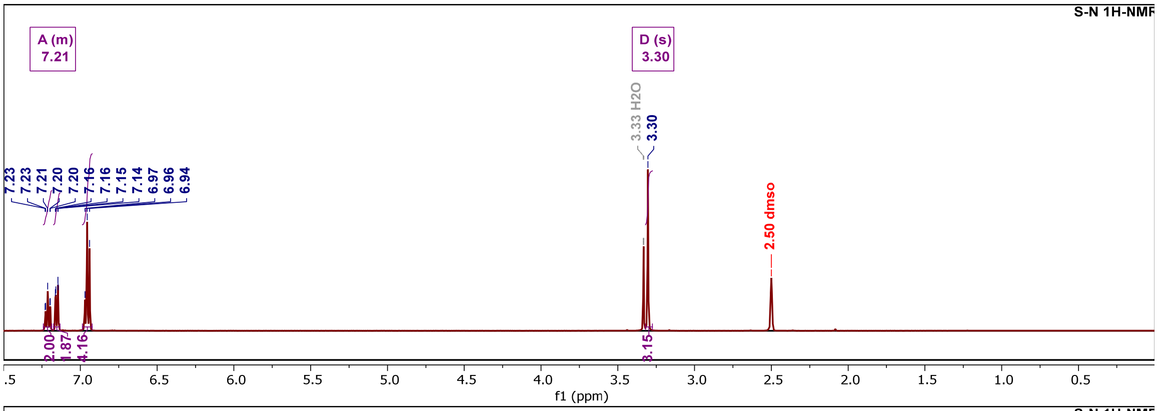

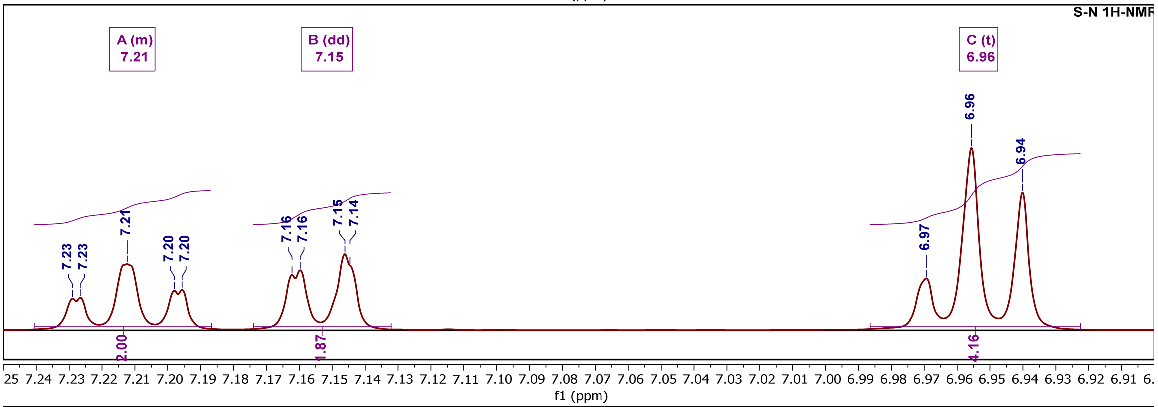

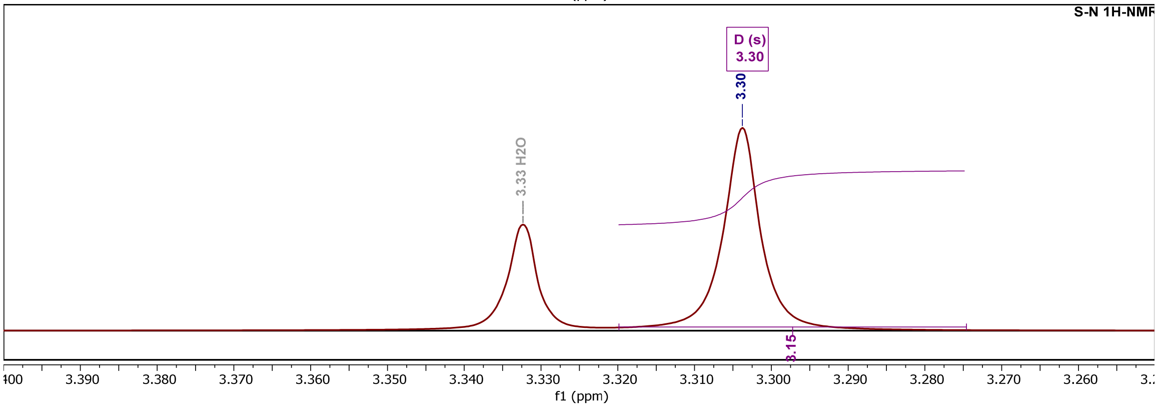

- **(*S*-N, 2) 10-methyl-10*H*-phenothiazine**

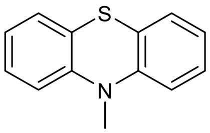

^{13}C NMR (126 MHz, DMSO-d6)

δ 145.78, 128.21, 127.22, 122.92, 122.53, 115.05, 35.56.

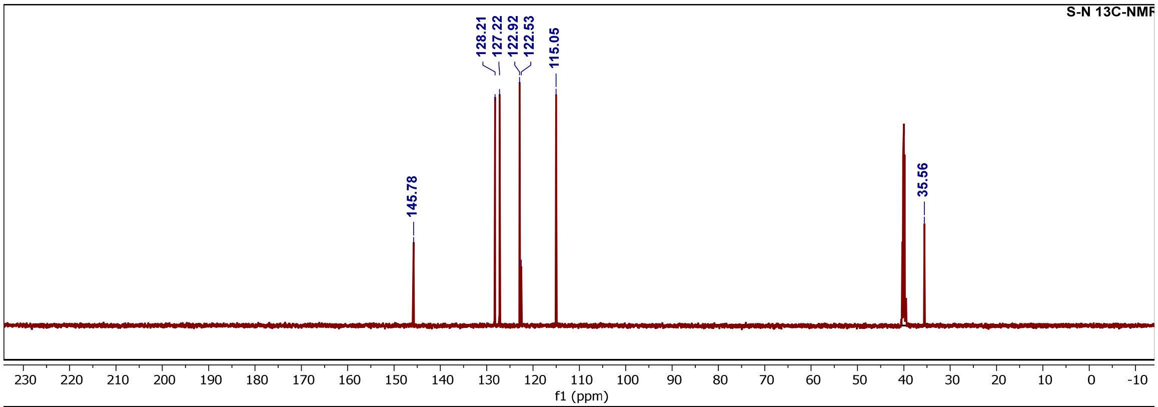

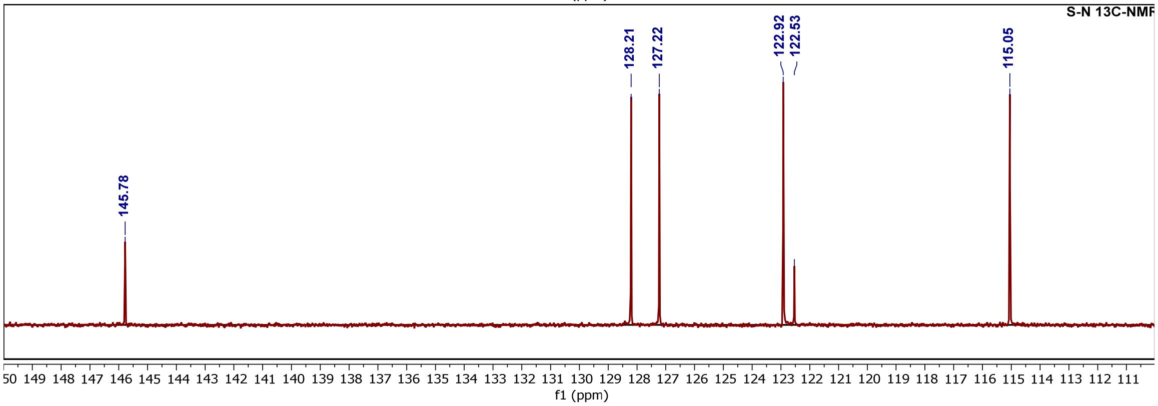

- **Dimethyl mesitylboronate, or MesB(OMe)$_2$**

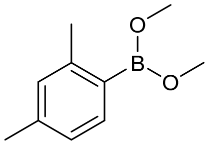

^{1}H NMR (400 MHz, Chloroform-d)

δ 6.81 (s, 2H), 3.56 (s, 5H), 2.32 – 2.20 (m, 9H).

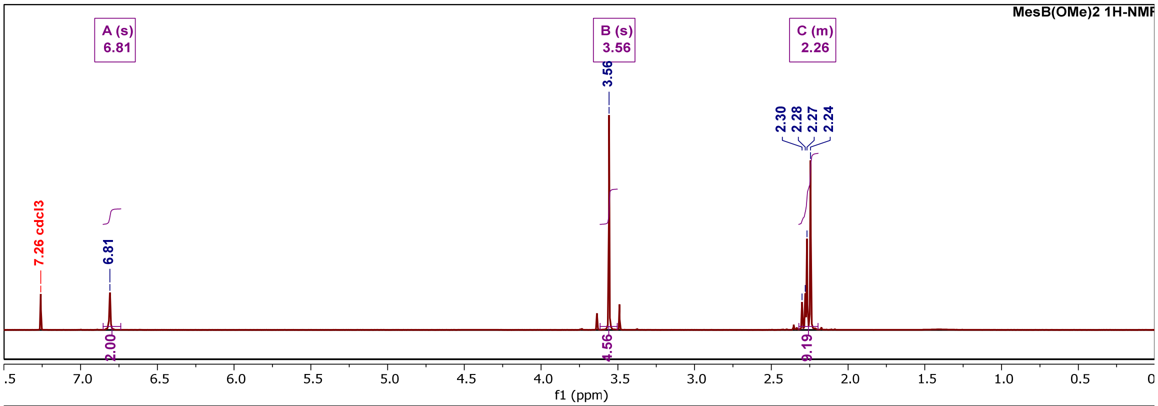

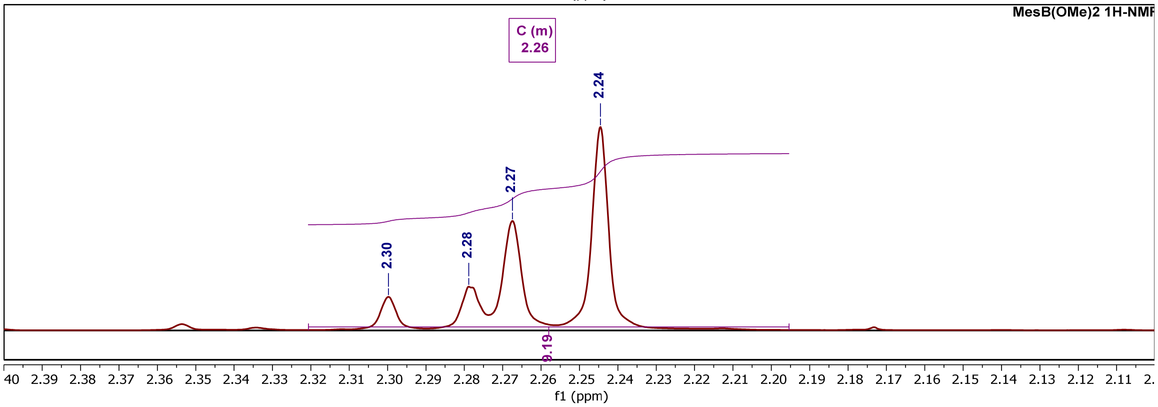

^{11}B NMR (128 MHz, Chloroform-d)

δ 31.30.

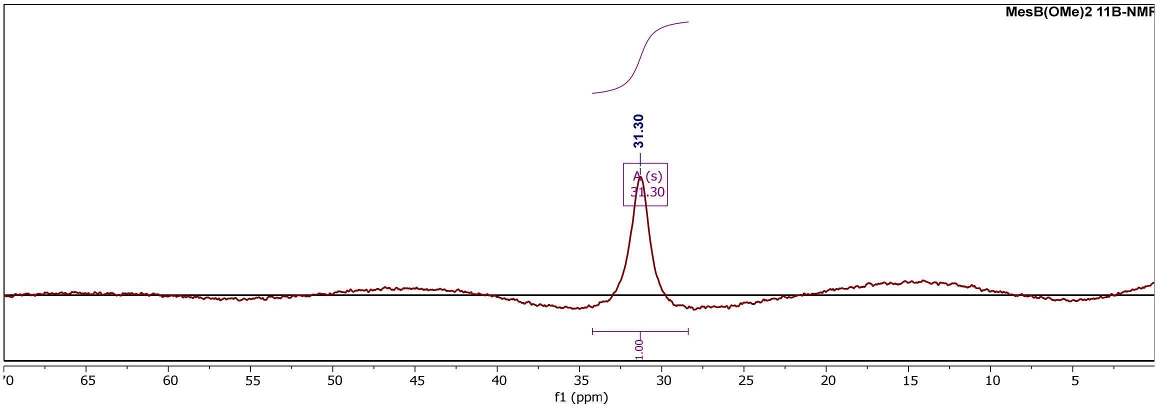

- **Bis(2-bromophenyl)selane**

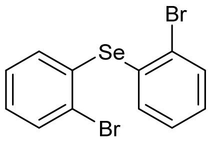

<u>*1H NMR (500 MHz, Chloroform-d)*</u>

δ 7.63 (dd, J = 7.6, 1.6 Hz, 2H), 7.25 (dd, J = 7.6, 1.9 Hz, 2H), 7.23 – 7.13 (m, 4H).

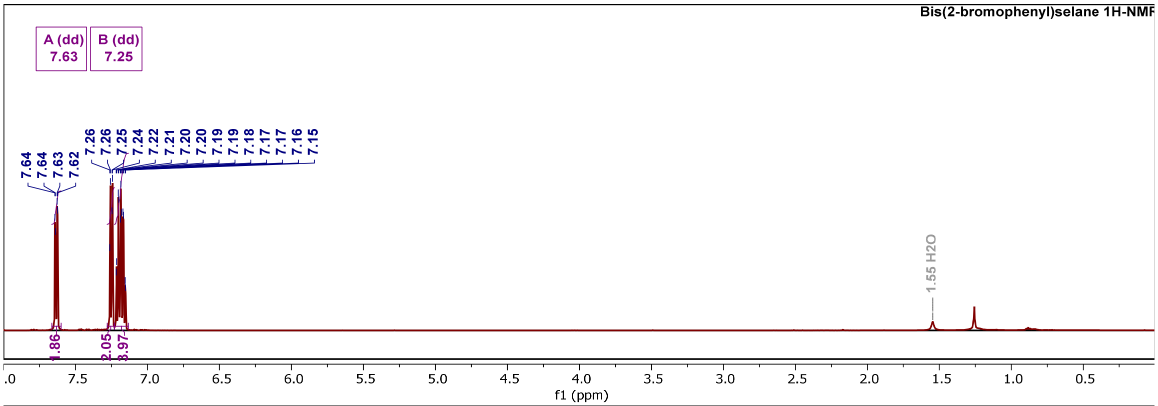

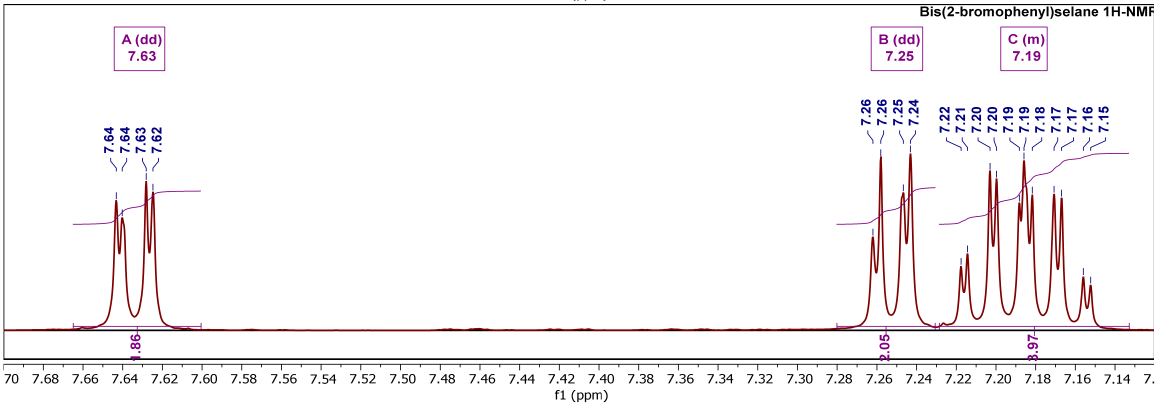

- **Bis(2-bromophenyl)selane**

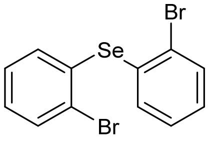

<u>*^{13}C NMR (126 MHz, DMSO-d6)*</u>

δ 134.44, 133.78, 132.71, 130.45, 129.44, 126.61.

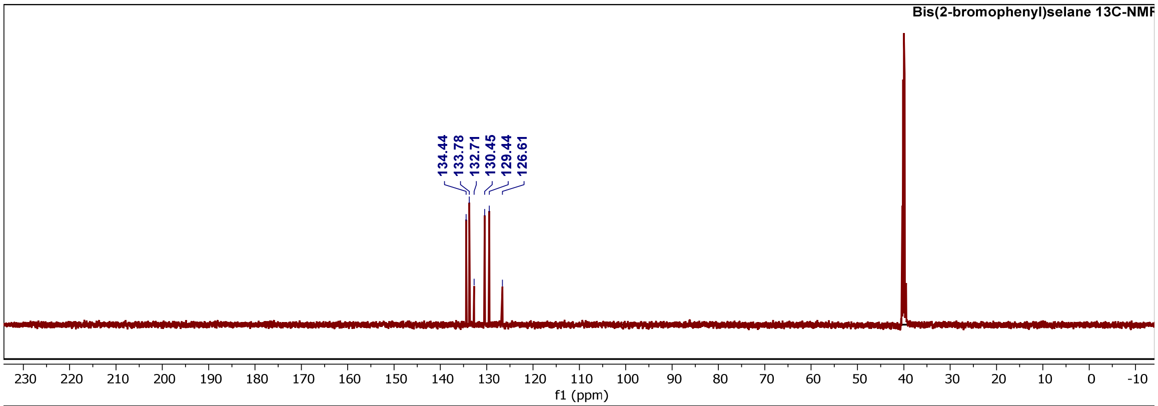

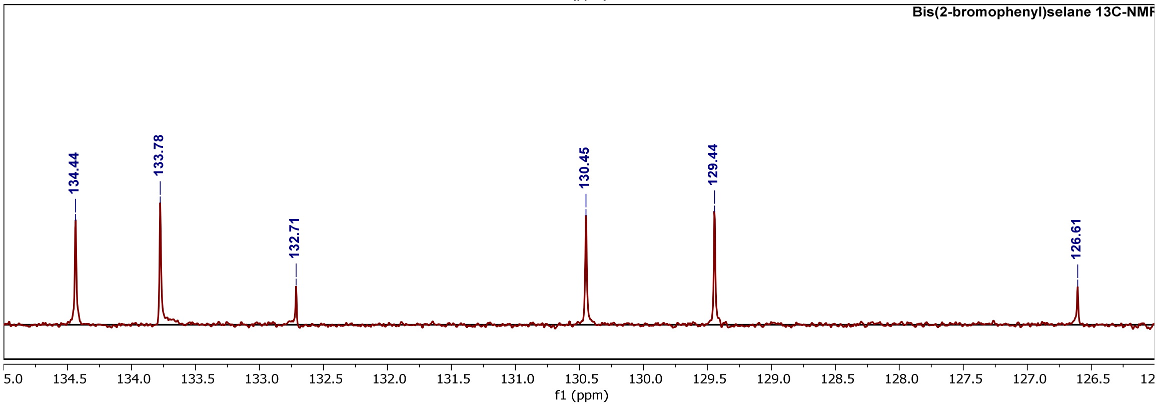

<u>*^{77}Se NMR (95 MHz, Chloroform-d)*</u>

δ 462.71.

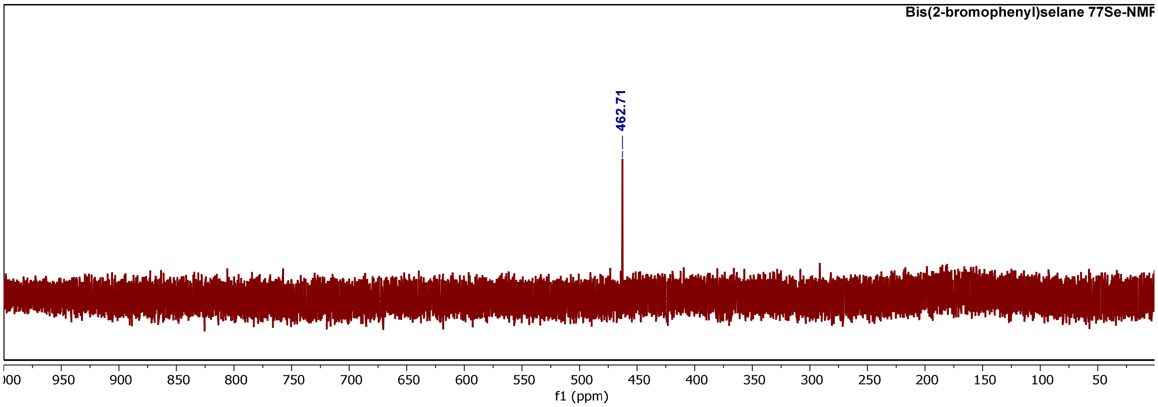

- **(*Se*-B, 5) 10-mesityl-10*H*-dibenzo[*b*,*e*][1,4]selenaborinine**

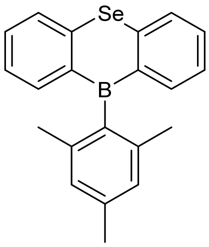

<u>^{1}H NMR (500 MHz, Chloroform-d)</u>

δ 7.92 – 7.87 (m, 1H), 7.84 (d, J = 7.9 Hz, 1H), 7.57 – 7.51 (m, 1H), 7.29 (t, J = 7.4 Hz, 1H), 6.94 (s, 1H), 2.41 (s, 2H), 1.93 (s, 3H).

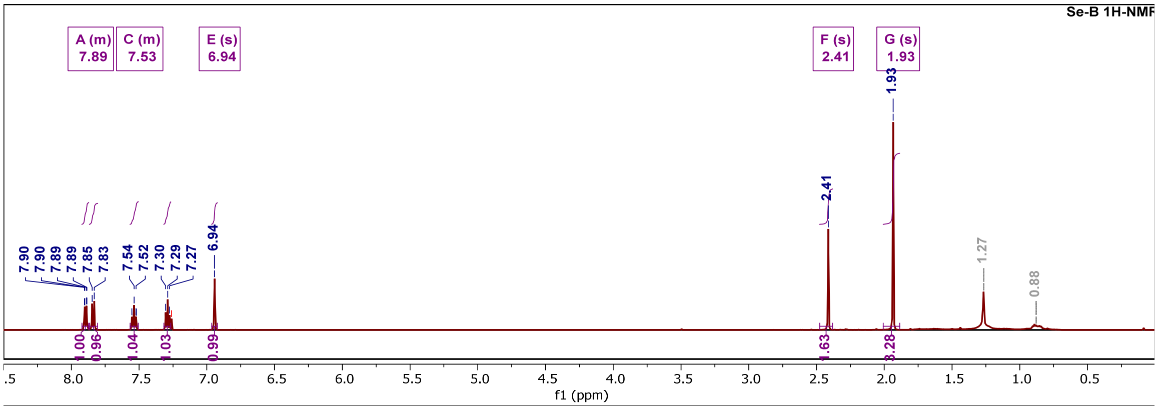

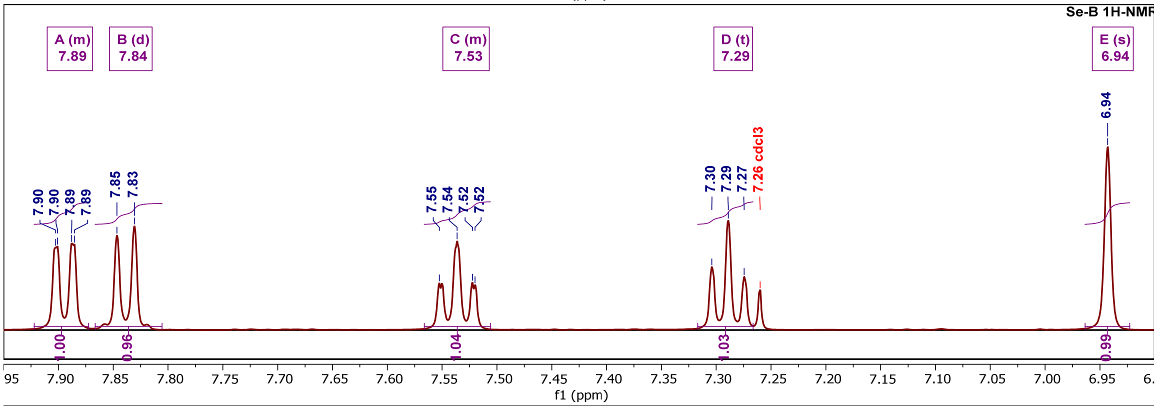

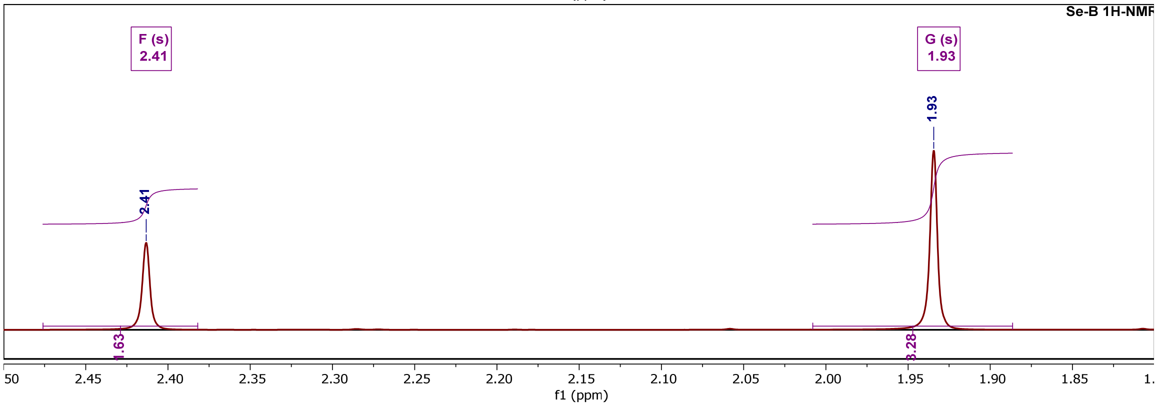

- **(*Se*-B, 5) 10-mesityl-10*H*-dibenzo[*b*,*e*][1,4]selenaborinine**

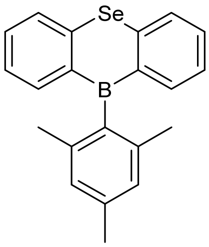

^{1}H NMR (401 MHz, DMSO-d6)

δ 8.01 (d, J = 7.6 Hz, 1H), 7.72 (dd, J = 7.7, 1.3 Hz, 1H), 7.69 – 7.61 (m, 1H), 7.41 – 7.34 (m, 1H), 6.92 (s, 1H), 2.33 (s, 2H), 1.83 (s, 3H).

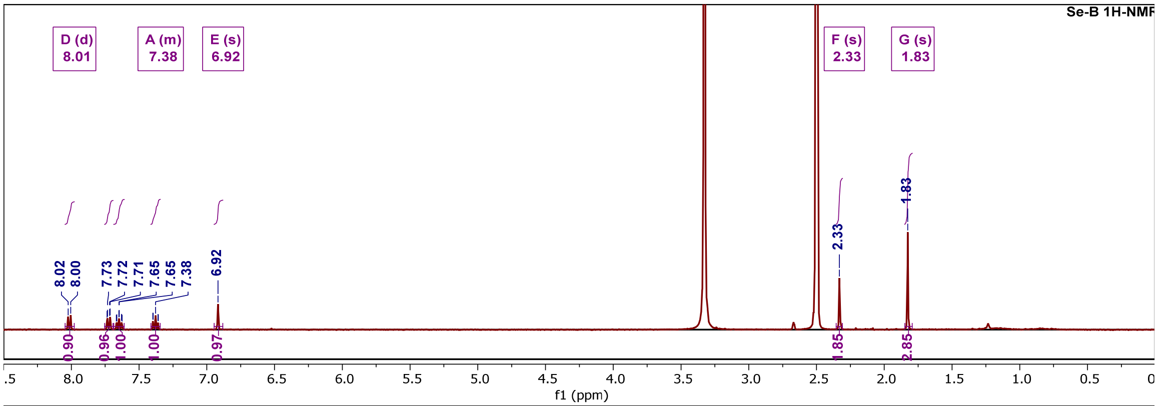

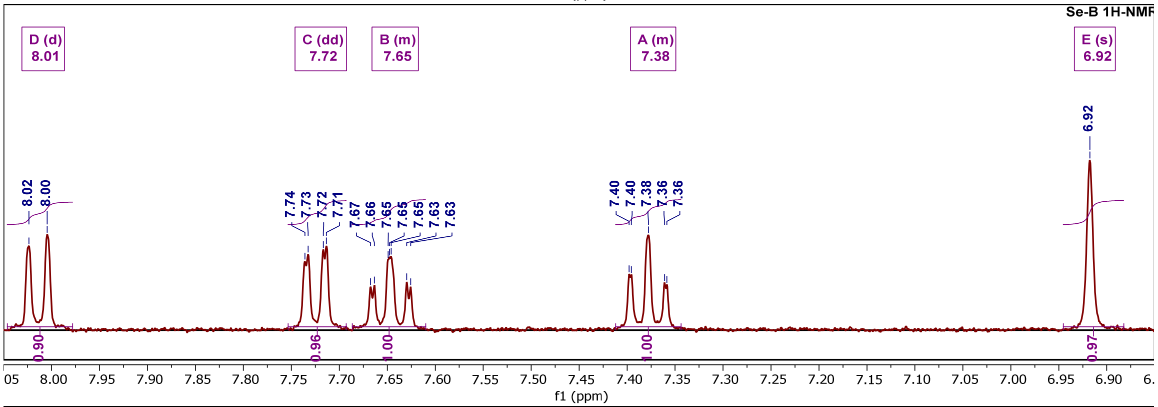

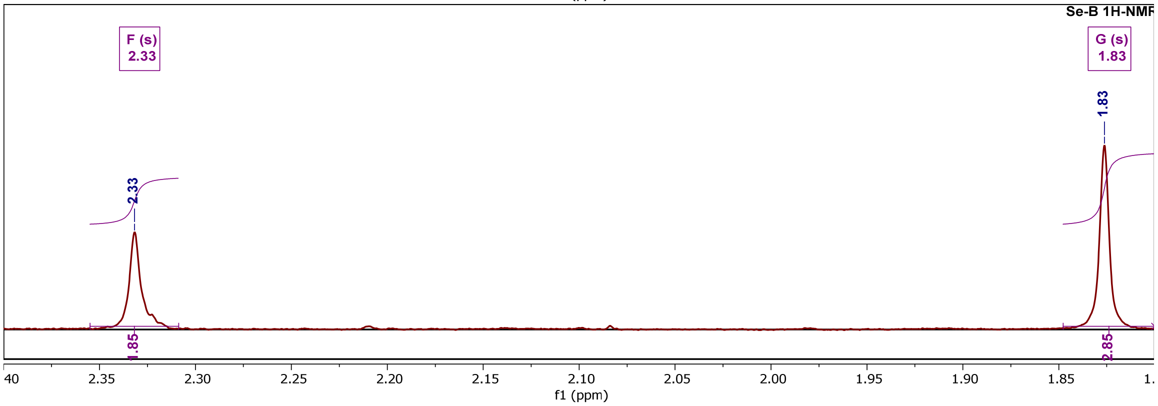

▪ **(Se-B, 5) 10-mesityl-10*H*-dibenzo[*b*,*e*][1,4]selenaborinine**

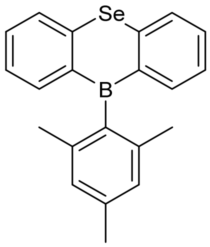

^{13}C NMR (126 MHz, DMSO-d6)

δ 143.74, 140.39, 137.46, 136.11, 134.96, 132.75, 127.86, 127.07, 125.71, 118.06, 22.50, 20.85.

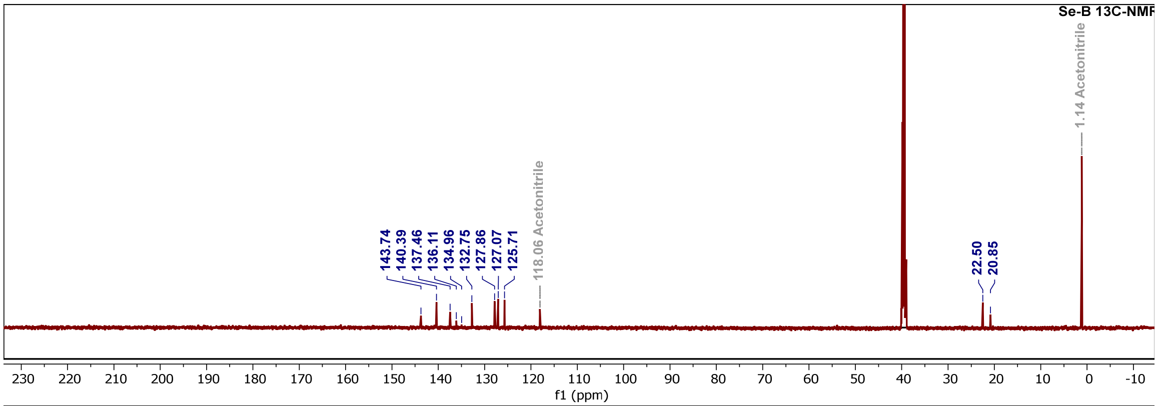

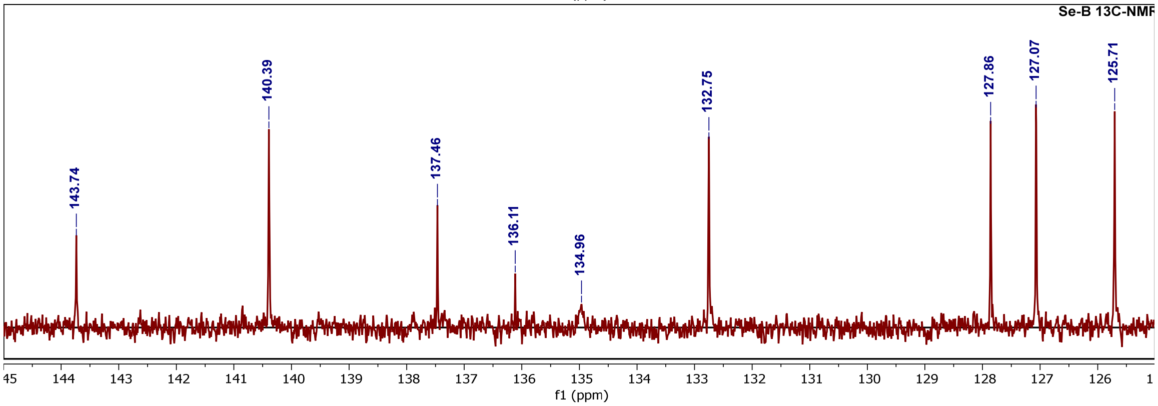

- **(Se-B, 5) 10-mesityl-10*H*-dibenzo[*b,e*][1,4]selenaborinine**

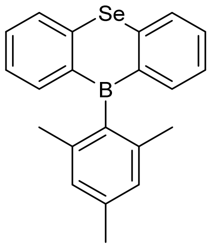

^{11}B NMR (128 MHz, Chloroform-d)

δ 61.79.

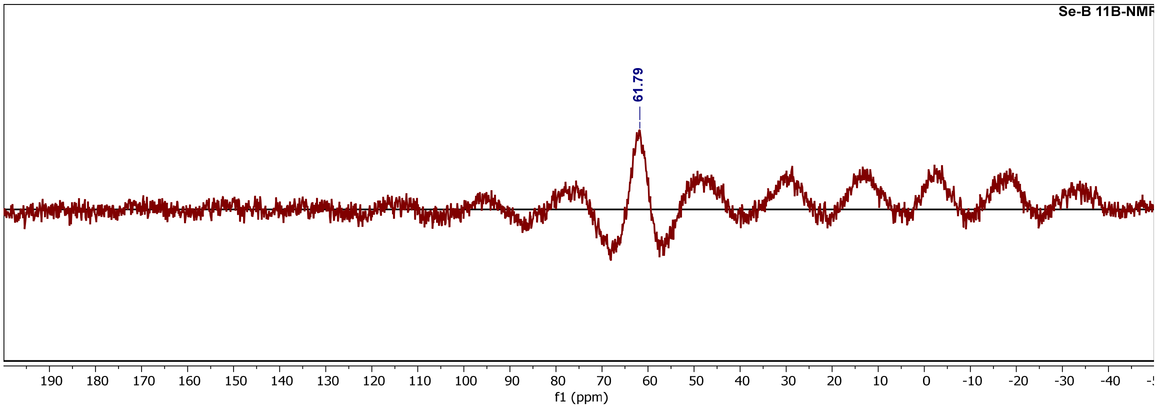

^{77}Se NMR (95 MHz, Chloroform-d)

δ 409.75.

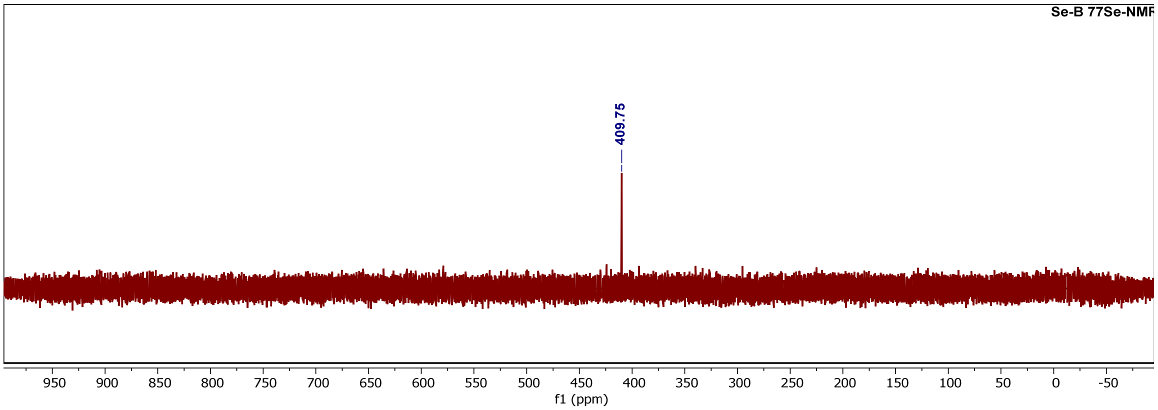

- **Bis(2-bromophenyl)sulfane**

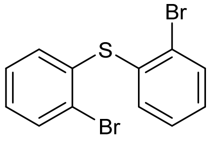

<u>^{1}H NMR (401 MHz, Chloroform-d)</u>

δ 7.65 (dd, J = 7.8, 1.2 Hz, 2H), 7.24 (dd, J = 7.6, 1.4 Hz, 2H), 7.18 – 7.11 (m, 4H).

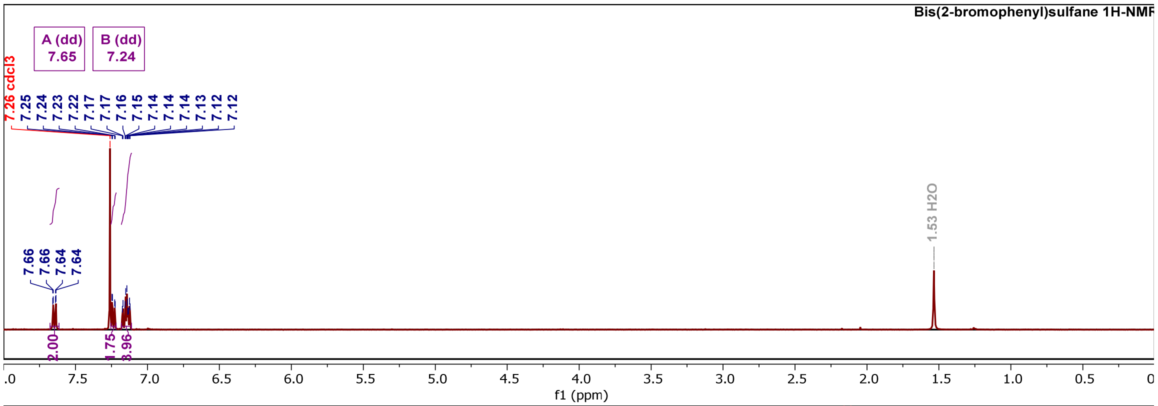

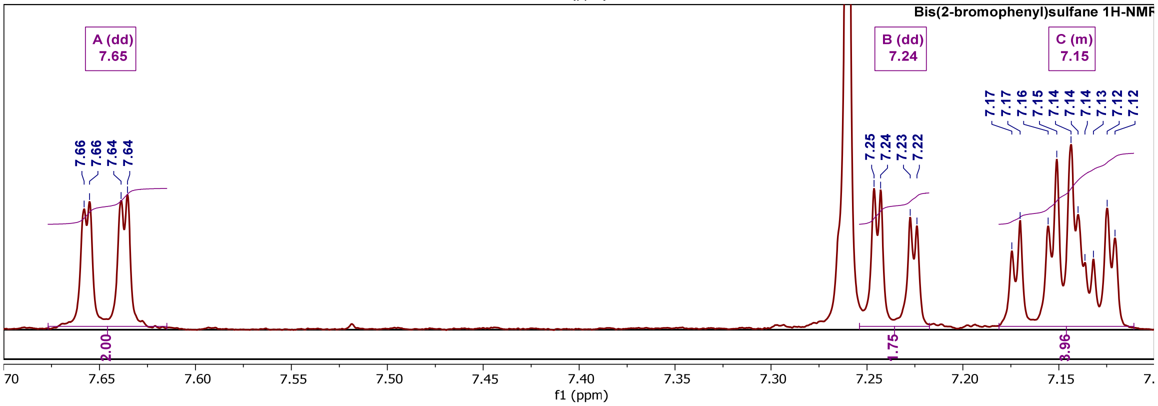

- **Bis(2-bromophenyl)sulfane**

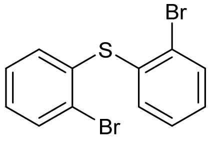

<u>*^1H NMR (500 MHz, DMSO-d6)*</u>

δ 7.79 – 7.75 (m, 2H), 7.42 – 7.38 (m, 2H), 7.30 (td, J = 7.6, 1.3 Hz, 2H), 7.13 (dd, J = 7.8, 1.4 Hz, 2H).

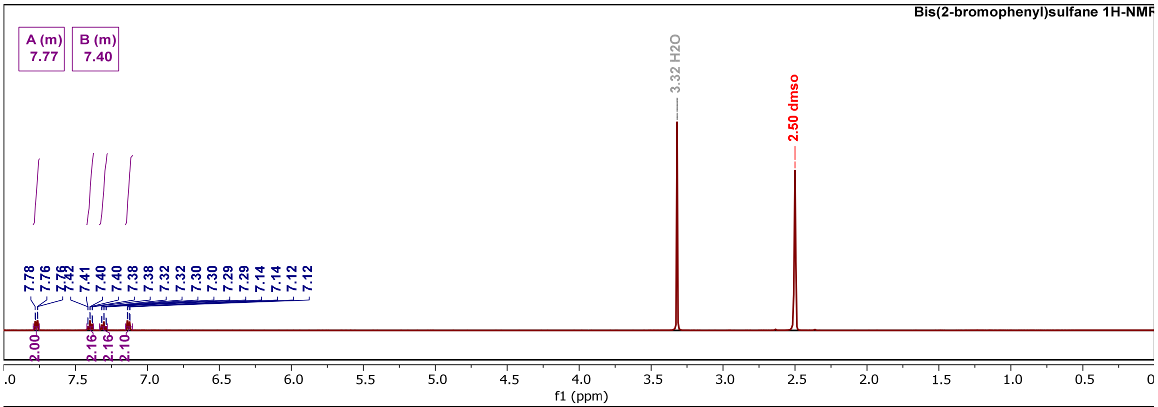

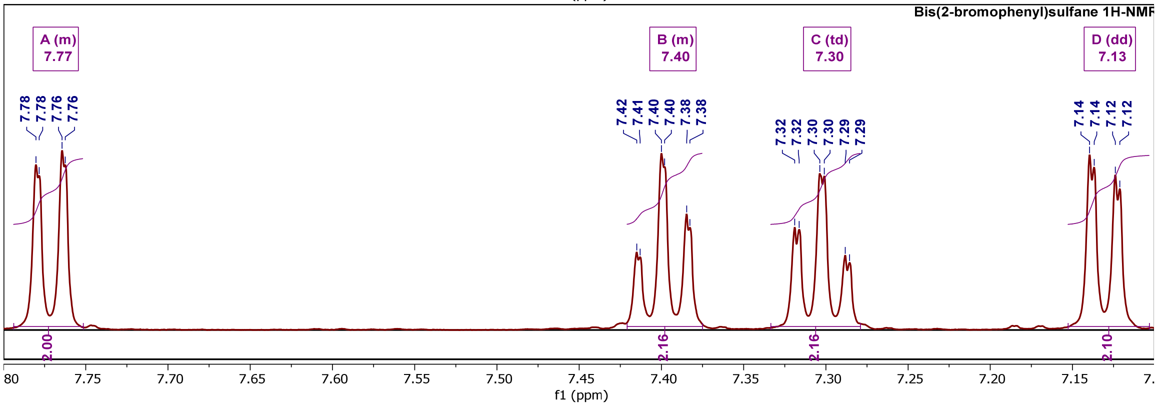

- **Bis(2-bromophenyl)sulfane**

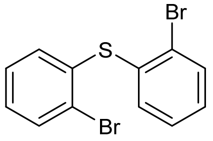

<u>*^{13}C NMR (126 MHz, DMSO-d6)*</u>

δ 134.81, 134.02, 132.68, 130.13, 129.43, 125.35.

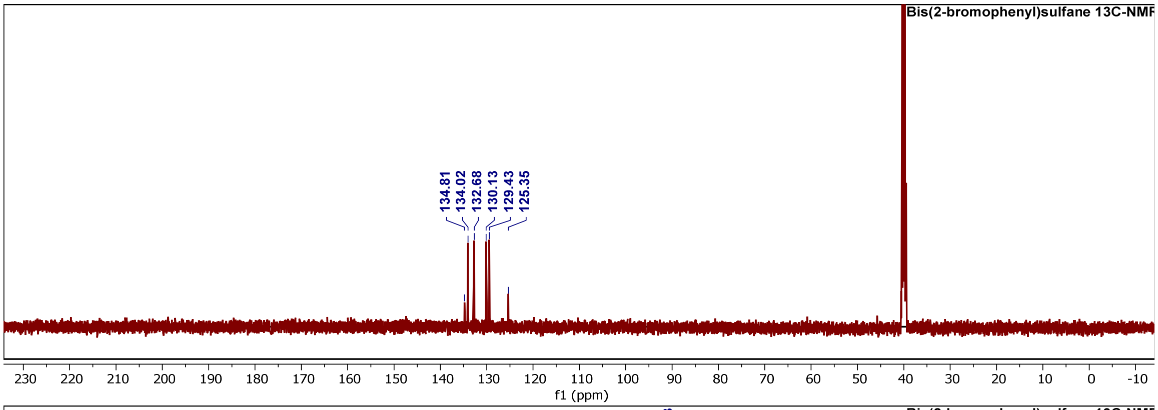

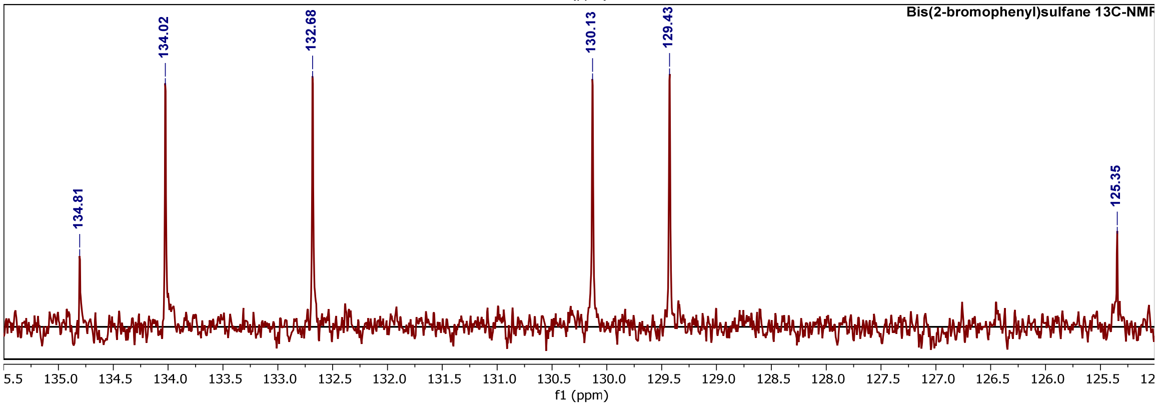

▪ (S-B, 6) 10-mesityl-10*H*-dibenzo[*b,e*][1,4]thiaborinine

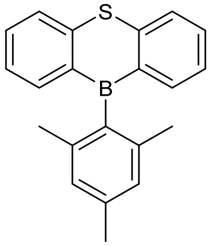

<u>¹H NMR (400 MHz, Chloroform-d)</u>

δ 7.88 (d, J = 7.7 Hz, 1H), 7.78 (d, J = 8.1 Hz, 1H), 7.62 (t, J = 7.5 Hz, 1H), 7.30 (t, J = 7.4 Hz, 1H), 6.95 (s, 1H), 2.41 (s, 2H), 1.93 (s, 3H).

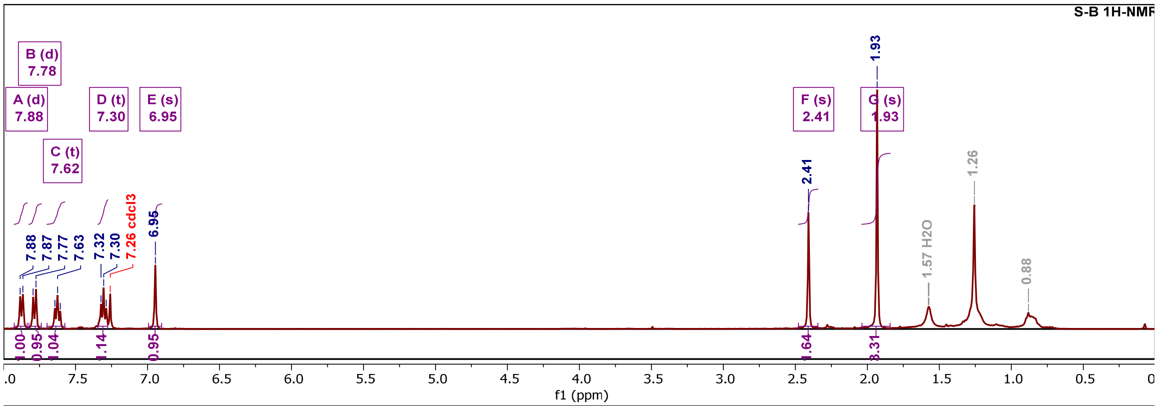

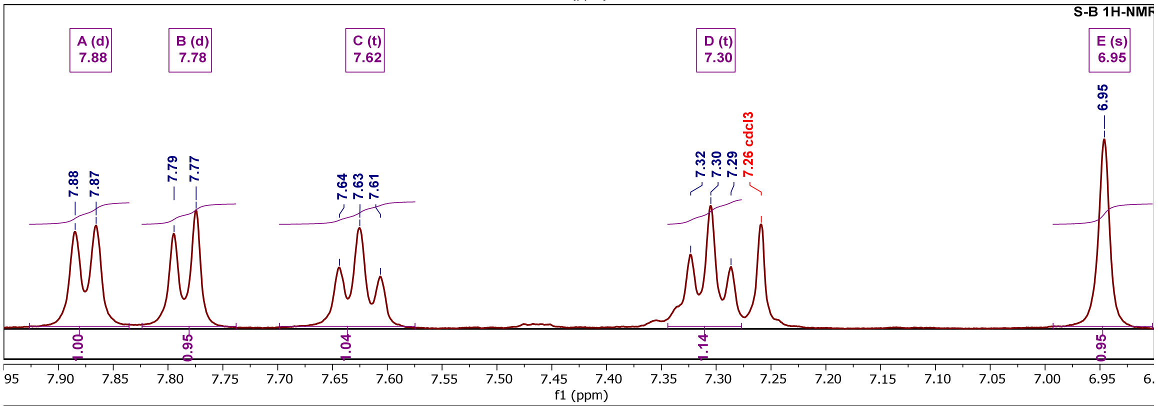

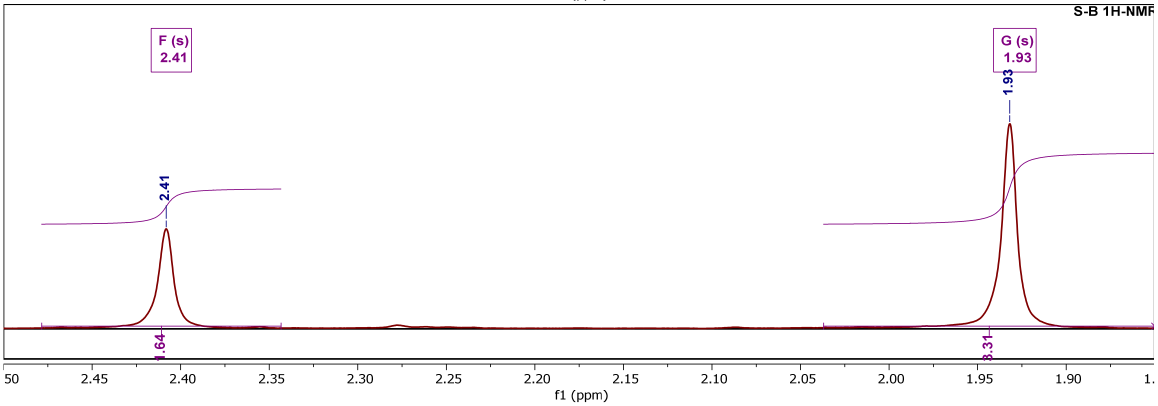

- **(S-B, 6) 10-mesityl-10*H*-dibenzo[*b*,*e*][1,4]thiaborinine**

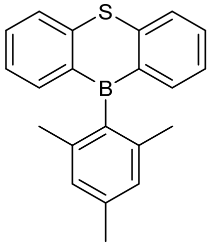

^{1}H NMR (500 MHz, DMSO-d6)

δ 7.94 (d, J = 8.1 Hz, 2H), 7.79 – 7.70 (m, 5H), 7.42 (t, J = 7.4 Hz, 2H), 6.94 (s, 2H), 2.34 (s, 3H), 1.83 (s, 6H).

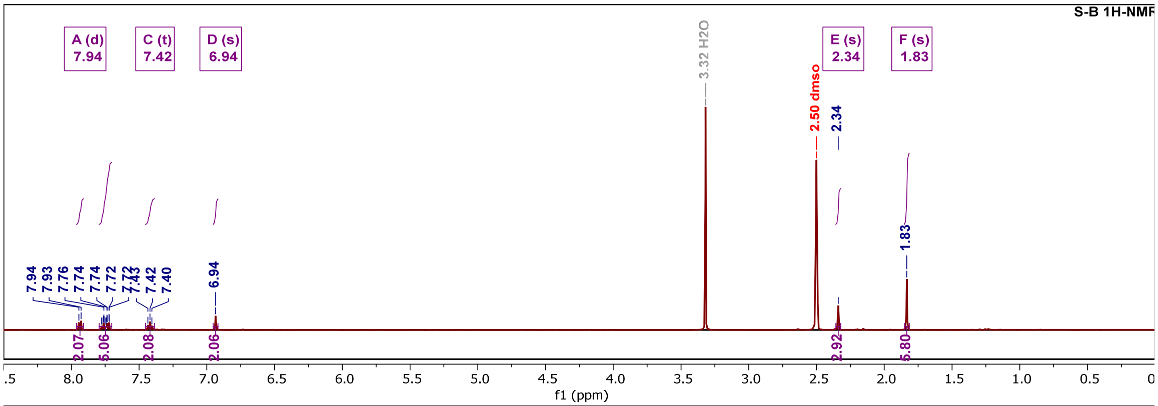

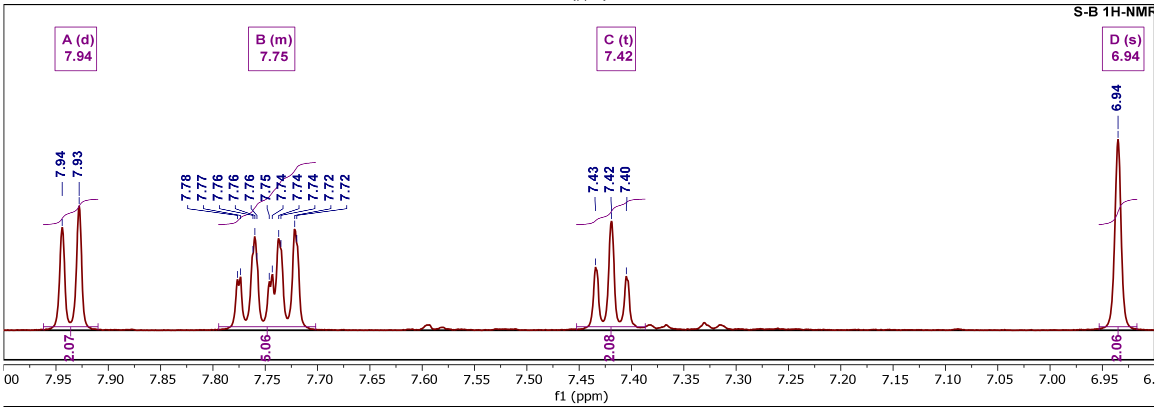

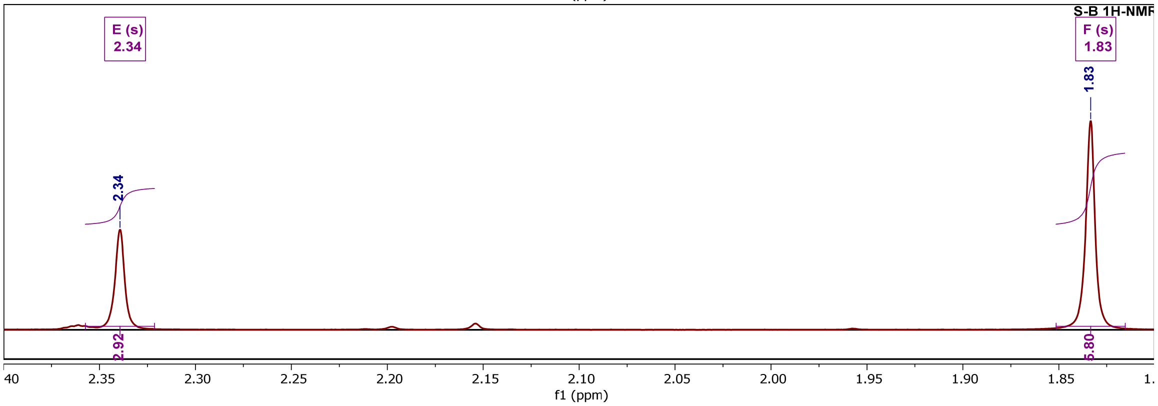

▪ **(S-B, 6) 10-mesityl-10*H*-dibenzo[*b,e*][1,4]thiaborinine**

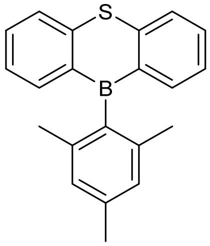

<u>*^{13}C NMR (126 MHz, DMSO-d6)*</u>

δ 143.90, 139.28, 138.10, 136.71, 133.42, 127.50, 126.04, 125.68, 23.00, 21.33.

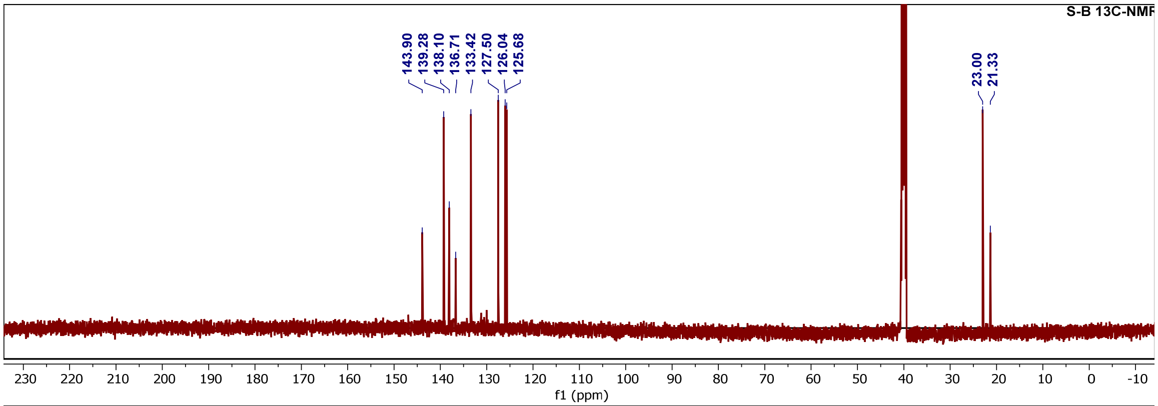

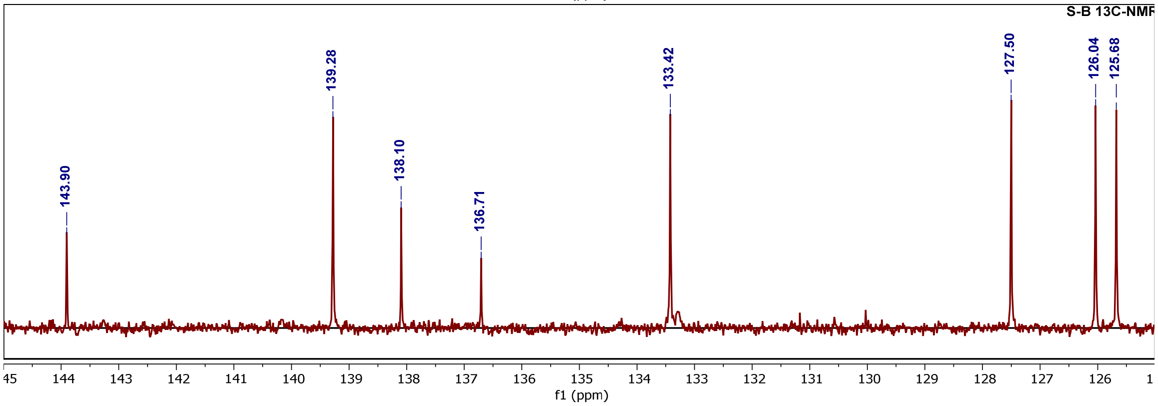

- **1,2-bis(2-phenoxyphenyl)diselane**

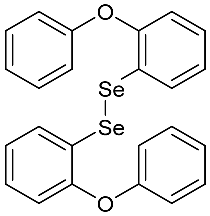

^{1}H NMR (700 MHz, Chloroform-d)

δ 7.69 (d, J = 7.9 Hz, 2H), 7.34 (t, J = 7.5 Hz, 4H), 7.18 (t, J = 7.7 Hz, 2H), 7.12 (t, J = 7.4 Hz, 2H), 7.02 (dd, J = 21.5, 8.0 Hz, 6H), 6.83 (d, J = 8.1 Hz, 2H).

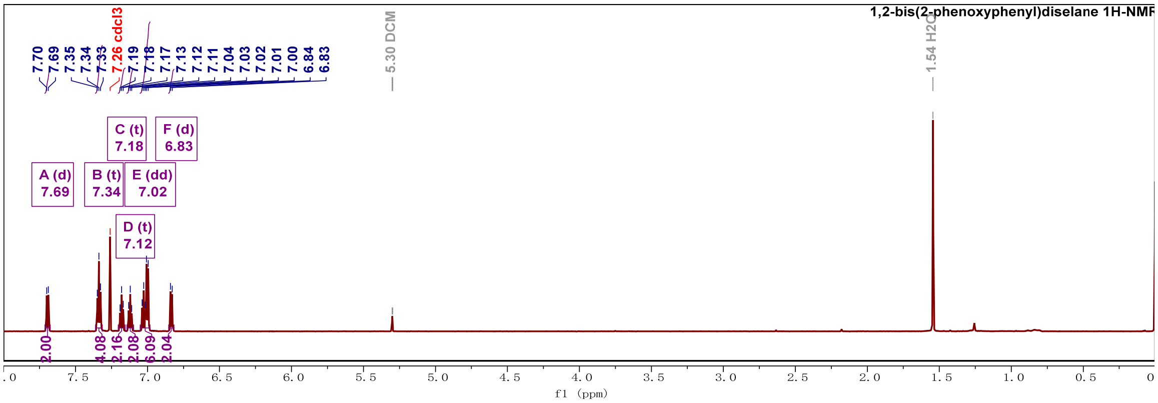

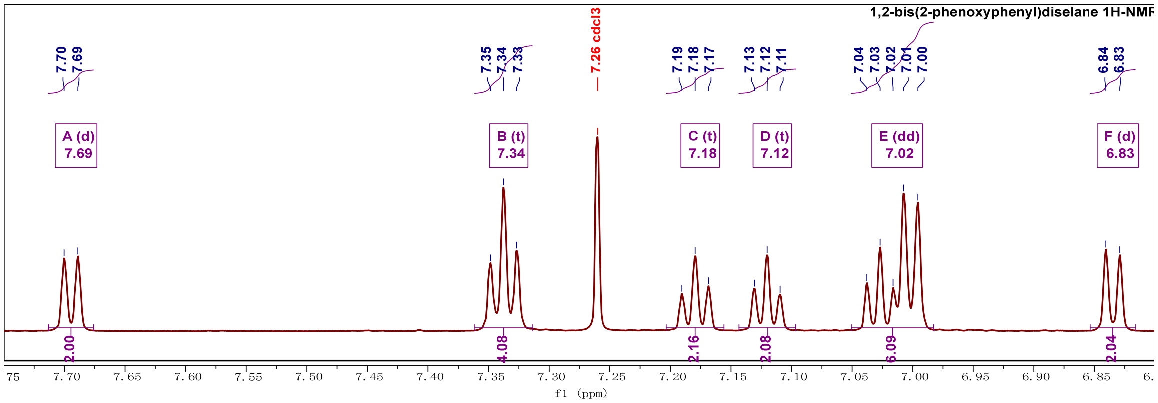

^{77}Se NMR (134 MHz, Chloroform-d)

δ 354.66.

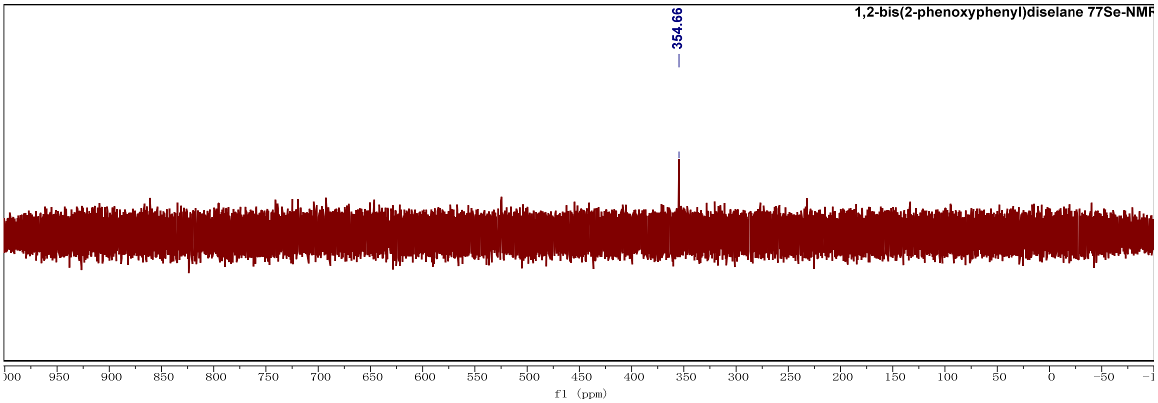

- **(Se-O, 3) Phenoxaselenine**

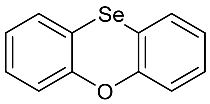

<u>*^{1}H NMR (500 MHz, Chloroform-d)*</u>

δ 7.30 (d, J = 7.6 Hz, 2H), 7.20 (t, J = 7.7 Hz, 2H), 7.12 (d, J = 8.0 Hz, 2H), 7.04 (t, J = 7.4 Hz, 2H).

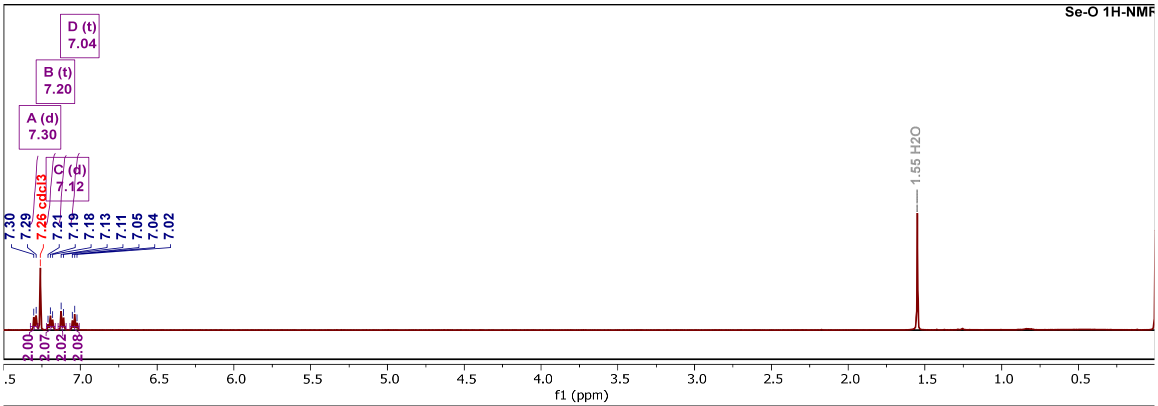

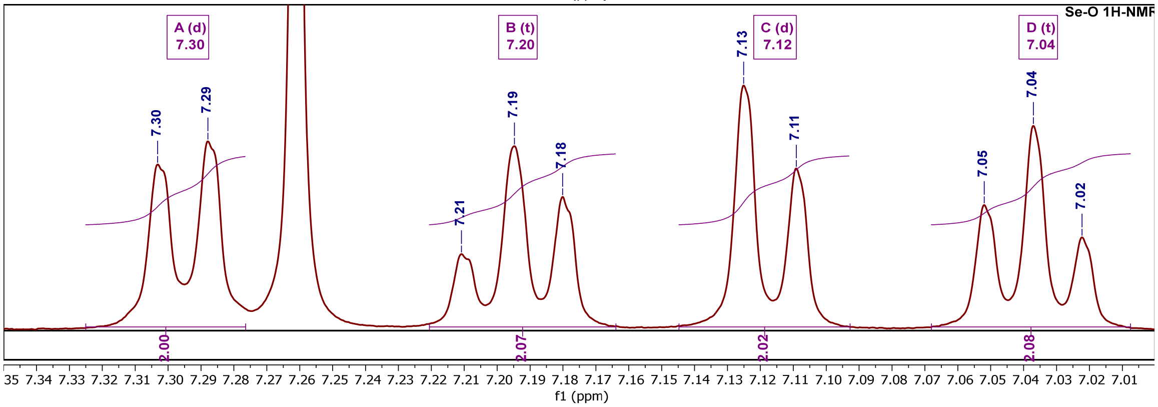

- **(Se-O, 3) Phenoxaselenine**

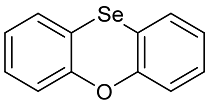

^{1}H NMR (500 MHz, DMSO-d6)

δ 7.47 (dd, J = 7.7, 1.4 Hz, 1H), 7.30 – 7.24 (m, 1H), 7.18 (dd, J = 8.1, 1.1 Hz, 1H), 7.12 (td, J = 7.5, 1.2 Hz, 1H).

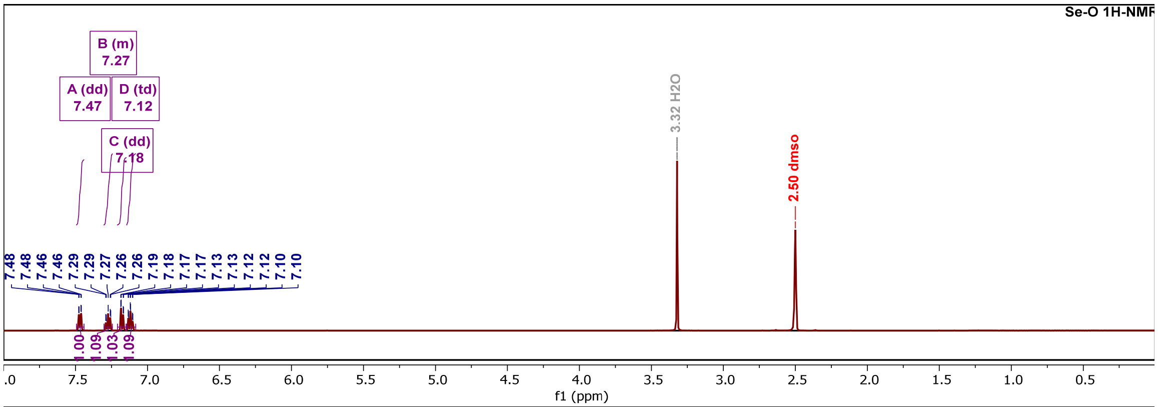

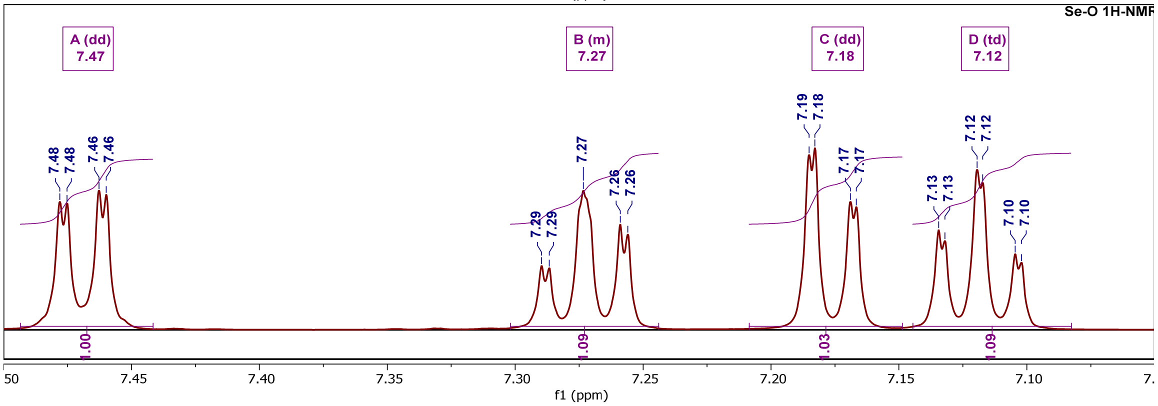

▪ **(Se-O, 3) Phenoxaselenine**

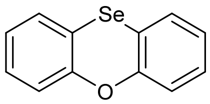

<u>^{13}C NMR (126 MHz, DMSO-d6)</u>

δ 152.68, 130.24, 129.11, 125.87, 118.96, 116.33.

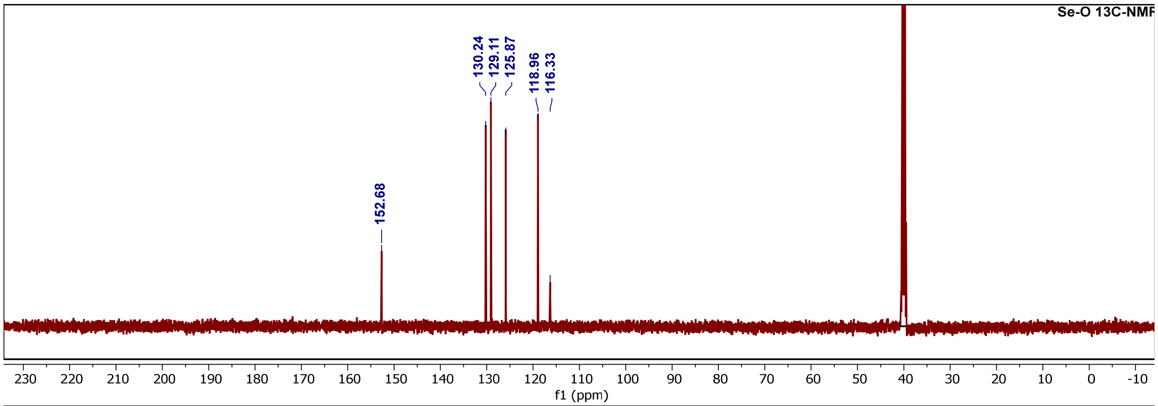

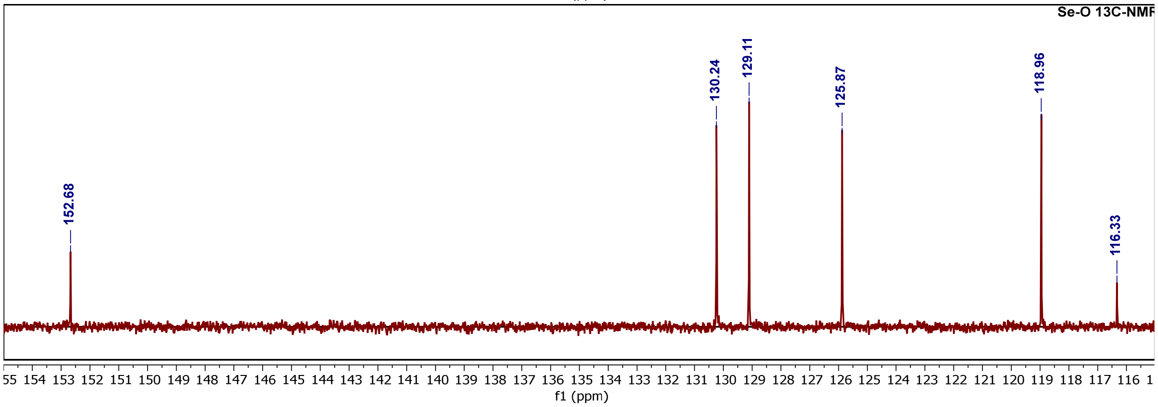

^{77}Se NMR (134 MHz, Chloroform-d)

δ 256.69.

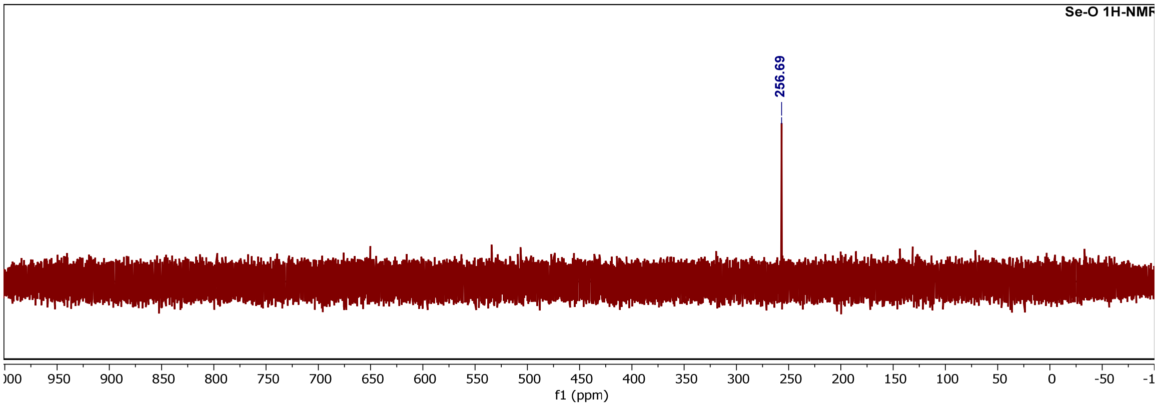

VII. Reference

1. Kolek, M., Otteny, F., Schmidt, P., Mück-Lichtenfeld, C., Einholz, C., Becking, J., Schleicher, E., Winter, M., Bieker, P. & Esser, B. Ultra-high cycling stability of poly(vinylphenothiazine) as a battery cathode material resulting from π-π Interactions. *Energy and Environmental Science* **10,** 2334–2341 (2017).
2. Kobayashi, J., Kato, K., Agou, T. & Kawashima, T. Synthesis of Dibenzochalcogenaborins and Systematic Comparisons of Their Optical Properties by Changing a Bridging Chalcogen Atom. *Chemistry - An Asian Journal* **4,** 42–49 (2009).
3. Toma, A. M., Nicoarə, A., Silvestru, A., Rüffer, T., Lang, H. & Mehring, M. Bis(2-Phenoxyphenyl)dichalcogenides and Their Chemical Reactivity. *Journal of Organometallic Chemistry* **810,** 33–39 (2016).
4. Ansari, R., Shao, W., Yoon, S. J., Kim, J. & Kieffer, J. Charge Transfer as the Key Parameter Affecting the Color Purity of Thermally Activated Delayed Fluorescence Emitters. *ACS Applied Materials and Interfaces* **13,** 28529–28537 (2021).
5. Knöller, J. A., Meng, G., Wang, X., Hall, D., Pershin, A., Beljonne, D., Olivier, Y., Laschat, S., Zysman-Colman, E. & Wang, S. Intramolecular Borylation via Sequential B−Mes Bond Cleavage for the Divergent Synthesis of B,N,B-Doped Benzo[4]helicenes. *Angewandte Chemie International Edition* **59,** 3156–3160 (2020).
6. Li, C., Mellerup, S. K., Wang, X. & Wang, S. Accessing Two-Stage Regioselective Photoisomerization in Unsymmetrical N,C-Chelate Organoboron Compounds: Reactivity of B(ppz)(Mes)Ar. *Organometallics* **37,** 3360–3367 (2018).
7. Yaqoob Bhat, M., Kumar, A. & Naveed Ahmed, Q. Selenium dioxide promoted dinitrogen extrusion/direct selenation of arylhydrazines and anilines. *Tetrahedron* **76,** 131105 (2020).
8. Li, Y., Nie, C., Wang, H., Li, X., Verpoort, F. & Duan, C. A Highly Efficient Method for the Copper-Catalyzed Selective Synthesis of Diaryl Chalcogenides from Easily Available Chalcogen Sources. *European Journal of Organic Chemistry* **2011,** 7331–7338 (2011).
9. Shao, Y., Gan, Z., Epifanovsky, E., Gilbert, A. T. B., Wormit, M., Kussmann, J., Lange, A. W., Behn, A., Deng, J., Feng, X., Ghosh, D., Goldey, M., Horn, P. R., Jacobson, L. D., Kaliman, I., Khaliullin, R. Z., Kuš, T., Landau, A., Liu, J., Proynov, E. I., Rhee, Y. M., Richard, R. M., Rohrdanz, M. A., Steele, R. P., Sundstrom, E. J., Woodcock, H. L., Zimmerman, P. M., Zuev, D., Albrecht, B., Alguire, E., Austin, B., Beran, G. J. O., Bernard, Y. A., Berquist, E., Brandhorst, K., Bravaya, K. B., Brown, S. T., Casanova, D., Chang, C. M., Chen, Y., Chien, S. H., Closser, K. D., Crittenden, D. L., Diedenhofen, M., Distasio, R. A., Do, H., Dutoi, A. D., Edgar, R. G., Fatehi, S., Fusti-Molnar, L., Ghysels, A., Golubeva-Zadorozhnaya, A., Gomes, J., Hanson-Heine, M. W. D., Harbach, P. H. P., Hauser, A. W., Hohenstein, E. G., Holden, Z. C., Jagau, T. C., Ji, H., Kaduk, B., Khistyaev, K., Kim, J., Kim, J., King, R. A., Klunzinger, P., Kosenkov, D., Kowalczyk, T., Krauter, C. M., Lao, K. U., Laurent, A. D., Lawler, K. v., Levchenko, S. v., Lin, C. Y., Liu, F., Livshits, E., Lochan, R. C., Luenser, A., Manohar, P., Manzer, S. F., Mao, S. P., Mardirossian, N., Marenich, A. v., Maurer, S. A., Mayhall, N. J., Neuscamman, E., Oana, C. M., Olivares-Amaya, R., Oneill, D. P., Parkhill, J. A., Perrine, T. M., Peverati, R., Prociuk, A., Rehn, D. R., Rosta, E., Russ, N. J., Sharada, S. M., Sharma, S., Small, D. W., Sodt, A., Stein, T., Stück, D., Su, Y. C., Thom, A. J. W., Tsuchimochi, T., Vanovschi, V., Vogt, L., Vydrov, O., Wang, T., Watson, M. A., Wenzel, J., White, A., Williams, C. F., Yang, J., Yeganeh, S., Yost, S. R., You, Z. Q., Zhang, I. Y., Zhang, X., Zhao, Y., Brooks, B. R., Chan, G. K. L., Chipman, D. M., Cramer, C. J., Goddard, W. A., Gordon, M. S., Hehre, W. J., Klamt, A., Schaefer, H. F., Schmidt, M. W., Sherrill, C. D., Truhlar, D. G., Warshel, A., Xu, X., Aspuru-Guzik, A., Baer, R., Bell, A. T.,

Besley, N. A., Chai, J. da, Dreuw, A., Dunietz, B. D., Furlani, T. R., Gwaltney, S. R., Hsu, C. P., Jung, Y., Kong, J., Lambrecht, D. S., Liang, W., Ochsenfeld, C., Rassolov, V. A., Slipchenko, L. v., Subotnik, J. E., van Voorhis, T., Herbert, J. M., Krylov, A. I., Gill, P. M. W. & Head-Gordon, M. Advances in Molecular Quantum Chemistry Contained in the Q-Chem 4 Program Package. *Molecular Physics* **113,** 184–215 (2015).

10. Weigend, F. Accurate Coulomb-fitting basis sets for H to Rn. *Physical Chemistry Chemical Physics* **8,** 1057–1065 (2006).
11. Weigend, F. & Ahlrichs, R. Balanced basis sets of split valence, triple zeta valence and quadruple zeta valence quality for H to Rn: Design and assessment of accuracy. *Physical Chemistry Chemical Physics* **7,** 3297–3305 (2005).
12. Chai, J. da & Head-Gordon, M. Systematic optimization of long-range corrected hybrid density functionals. *Journal of Chemical Physics* **128,** 84106 (2008).
13. Chai, J. da & Head-Gordon, M. Long-range corrected hybrid density functionals with damped atom-atom dispersion corrections. *Physical Chemistry Chemical Physics* **10,** 6615–6620 (2008).
14. Pokhilko, P., Epifanovsky, E. & Krylov, A. I. General framework for calculating spin-orbit couplings using spinless one-particle density matrices: Theory and application to the equation-of-motion coupled-cluster wave functions. *Journal of Chemical Physics* **151,** (2019).
15. Plasser, F., Wormit, M. & Dreuw, A. New tools for the systematic analysis and visualization of electronic excitations. I. Formalism. *Journal of Chemical Physics* **141,** 024106 (2014).
16. Allouche, A.-R. Gabedit-A graphical user interface for computational chemistry softwares. *Journal of Computational Chemistry* **32,** 174–182 (2011).
